# Taurine Inhibits Ferroptosis Mediated by the Crosstalk between Tumor Cells and Tumor‐Associated Macrophages in Prostate Cancer

**DOI:** 10.1002/advs.202303894

**Published:** 2023-11-29

**Authors:** Huixiang Xiao, Xinxing Du, Zhenkeke Tao, Nan Jing, Shijia Bao, Wei‐Qiang Gao, Baijun Dong, Yu‐Xiang Fang

**Affiliations:** ^1^ State Key Laboratory of Systems Medicine for Cancer Renji‐Med X Clinical Stem Cell Research Center Department of Urology Ren Ji Hospital School of Medicine Shanghai Jiao Tong University Shanghai 200127 P. R. China; ^2^ Department of Urology Ren Ji Hospital School of Medicine Shanghai Jiao Tong University Shanghai 200127 P. R. China; ^3^ School of Biomedical Engineering and Med‐X Research Institute Shanghai Jiao Tong University Shanghai 200030 P. R. China

**Keywords:** extracellular vesicles, ferroptosis, prostate cancer, taurine, tumor‐associated macrophages

## Abstract

Tumor‐associated macrophages (TAMs) play an essential role in tumor therapeutic resistance. Although the lethal effect of ferroptosis on tumor cells is well reported, how TAMs inhibit the effect of ferroptosis in tumors has not been clearly defined. In this study, it is demonstrated that TAM‐secreted taurine suppresses ferroptosis in prostate cancer (PCa) by activating the Liver X receptor alpha/Stearoyl‐Coenzyme A desaturase 1 (LXRα/SCD1) pathway. Blocking taurine intake via inhibition of taurine transporter TauT restores the sensitivity to ferroptosis in tumors. Furthermore, LXRα activates the transcription of both miR‐181a‐5p and its binding protein FUS to increase the recruitment of miR‐181a‐5p in tumor‐derived extracellular vesicles (EVs). It is observed that macrophages appear to be recipient cells of the miR‐181a‐5p‐enriched EVs. Intake of miR‐181a‐5p in macrophages promotes their M2 polarization and enhances the taurine export by inhibiting expression of its target gene lats1, which in turn inactivates the hippo pathway and results in a Yes‐associated protein (YAP) nuclear translocation for transcriptional activation of both M2 polarization‐related genes such as ARG1 and CD163 and the taurine transport gene TauT. Taken together, the findings indicate a reciprocal interaction between PCa cells and TAMs as a positive feedback‐loop to repress ferroptosis in PCa, mediated by TAM‐secreted taurine and tumor EV‐delivered miR‐181a‐5p.

## Graphical abstract

Taurine inhibits ferroptosis mediated by the crosstalk between tumor cells and TAMs via activation of LXRα/SCD1 axis in PCa. LXRα promotes miR‐181a‐5p expression/recruitment into tumor‐released EVs. Intake of miR‐181a‐5p induces M2 polarization in macrophages to enhance taurine export as a positive feedback loop. Targeting TauT‐mediated taurine transport is a promising strategy to restore the sensitivity to ferroptosis during PCa treatment.

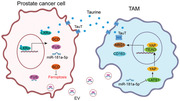

## 1 Introduction

It has been well‐reported that infiltration of macrophages that are defined as tumor‐associated macrophages (TAMs) in the tumor microenvironment are tightly related to the resistance of tumor therapy.^[^ ^1^ ^]^ In prostate cancer (PCa), TAMs are major immune cells in the tumor microenvironment to facilitate PCa progression via enhancing PCa cell migration and metastasis, promoting drug resistance, and so on.^[^ ^2^, ^3^, ^4^, ^5^, ^6^ ^]^ Functionally, TAMs are primarily composed of M2‐type macrophages, which can be polarized by various tumor microenvironmental factors such as chemokines, cytokines and tumor‐derived extracellular vesicles (EVs).^[^ ^7^, ^8^ ^]^

Currently, besides their promoting effects on resistance of chemotherapy and immunotherapy, TAMs are reported to be also involved in the resistance of ferroptosis by secretion of transforming growth factor beta 1.^[^ ^9^ ^]^ Ferroptosis is a programmed cell death characterized by the accumulation of lipid peroxidation and iron, eventually causing the rupture of membrane.^[^ ^10^ ^]^ It is a main mechanism for anti‐ferroptosis by activation of Stearoyl‐Coenzyme A desaturase 1 (SCD1) to produce monounsaturated fatty acids (MUFAs) for blocking the lipid ROS accumulation on the plasma membrane.^[^ ^11^, ^12^ ^]^ For example, mTORC1‐dependent activation of SREBP1 promotes the transcription of SCD1 for suppression of ferroptosis in PCa.^[^ ^13^ ^]^ Besides dysregulation of upstream pathways in tumors, factors such as cytokines and metabolites from microenvironment also play an important role in the activation of SCD1.^[^ ^14^, ^15^ ^]^ Recently, lactate secreted from cancer‐associated fibroblasts (CAFs) has been reported to be absorbed by liver cancer cells to suppress ferroptosis via activating the HCAR1/MCT1‐SREBP1‐SCD1 axis.^[^ ^16^ ^]^ These findings provide reasons for us to speculate whether macrophages can also secrete certain metabolites to promote the resistance of ferroptosis by activating SCD1 in PCa.

On the other hand, tumor secreta including free factors and EVs are major messengers to induce the M2 polarization of tumor‐infiltrated macrophages so to form an oncogenic microenvironment.^[^ ^17^, ^18^ ^]^ These M2 macrophages can then export proteins or metabolites that can then act on tumor cells to promote tumor progression and treatment resistance.^[^ ^19^ ^]^ For example, tumor‐derived EVs in which microRNAs and lncRNAs are enriched can promote M2 polarization in macrophages to increase the secretion of specific molecules to influence therapeutic resistance in tumors.^[^ ^17^, ^20^ ^]^ These studies indicate that the tumor cell‐macrophage crosstalk plays an important role in treatment resistance in tumors. However, what is the exact mechanism and what molecules, EVs or metabolites mediating such crosstalk are still unclear.

In this study, we screened and identified TAM‐secreted taurine as an available metabolite to promote the resistance of ferroptosis in PCa in a SCD1‐dependent manner. We found that intake of miR‐181a‐5p‐enriched EVs derived from PCa cells into microphages promoted their M2 polarization to increase taurine secretion as feedback. Our findings indicate that blockage of taurine export/import to disrupt the tumor cell‐macrophage crosstalk might be a potential strategy to restore the sensitivity to ferroptosis in PCa.

## 2 Results

### 2.1 Tumor Infiltrated M2 Type Macrophages were Involved in an Inhibition of Ferroptosis in PCa

**Figure 1:**
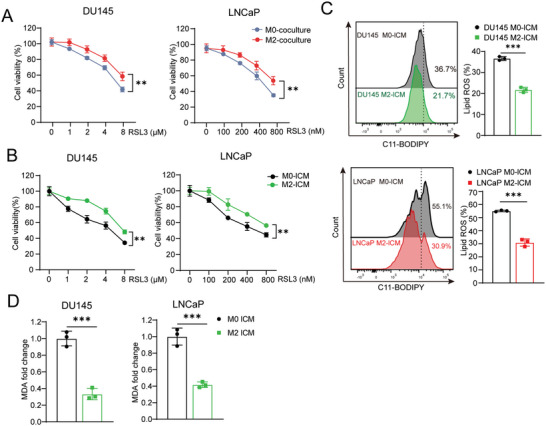
Low molecular weight components in M2 macrophage supernatant exhibit potent ferroptosis‐inhibiting properties in prostate cancer cells. A,B) Cell viability of PCa cells was checked after co‐culture with THP1‐derived M0/M2 macrophage (A) or incubated with M0‐lCM or M2‐lCM (B) for 24 h following with the RSL3 treatment for another 24 h. C,D) Lipid peroxidation (C) and MDA level (D) of PCa cells was measured after incubated with M0‐lCM or M2‐lCM for 24 h following with the RSL3 treatment for another 24 h. Each experiment was performed in triplicate and independently repeated three times. (Two‐tailed Student's *t*‐test was used for the statistical analysis: ^**^, *p* < 0.01; ^***^, *p* < 0.001. Data are presented as means ± SD, *n* = 3).

In order to evaluate the function of M2 type macrophages on prevention of PCa from ferroptosis, THP‐1‐derived M0 type macrophages (Figure S1A,B, Supporting Information) were first induced to become M2 macrophages, which was confirmed by upregulated expression of M2 macrophage markers including ARG1, CD163, and IL‐10 (Figure S1C,D, Supporting Information). Then, M0 or M2 macrophages were co‐cultured with LNCaP and DU‐145 cells respectively. After removing macrophages and replating PCa cells, ferroptosis inducer RSL3 was added into the culture for 24 h and a higher survival rate was observed in the M2 macrophage‐PCa cell co‐culture group than the M0 macrophage‐PCa cell co‐culture group, indicating the involvement of M2 macrophages in the inhibition of ferroptosis in PCa (**Figure** **1**A; Figure S1E, Supporting Information). Furthermore, M0 or M2 macrophage culture medium was termed as M0 or M2 conditional medium, respectively (i.e., M0 CM or M2 CM) and used to culture PCa cells for 12 h. After the conditional medium was replaced with fresh medium and followed with a ferroptosis inducer (RSL3 or Erastin) treatment for 24 h, we found that pre‐treatment with M2 CM significantly improved the survival rate of LNCaP cells, implying that the M2 macrophage‐conditioned medium contained functional factors such as secretory proteins or metabolite to promote the resistance of ferroptosis in PCa (Figure S1F,G, Supporting Information). For the purpose of identifying the effective components secreted by macrophages for anti‐ferroptosis, M0 CM or M2 CM was filtrated to separate the high molecular weight components (>10 KDa, with secretory proteins and EVs as major contents) and low molecular weight components (<10 KDa, with metabolite as major contents), respectively. Basic medium supplemented with high molecular weight components (named M0‐hCM and M2‐hCM respectively) or low molecular weight components (named M0‐lCM and M2‐lCM respectively) was used for pre‐treatment before inducing ferroptosis in PCa cells. As shown in Figure S1H (Supporting Information), we found that either M0‐hCM or M2‐hCM showed any inhibiting effects on ferroptosis. However, M2‐lCM but not M0‐lCM exhibited a significant inhibitory effect on ferroptosis based on assays of cell viability, lipid ROS and MDA (Figure 1B–D). Taken together, these results indicated that the low molecular weight components secreted by M2 macrophages, such as metabolite, might be taken in by PCa and were responsible for the inhibition of ferroptosis.

### 2.2 M2 Macrophages Produce Taurine to Repress Ferroptosis in Prostate Cancer

**Figure 2:**
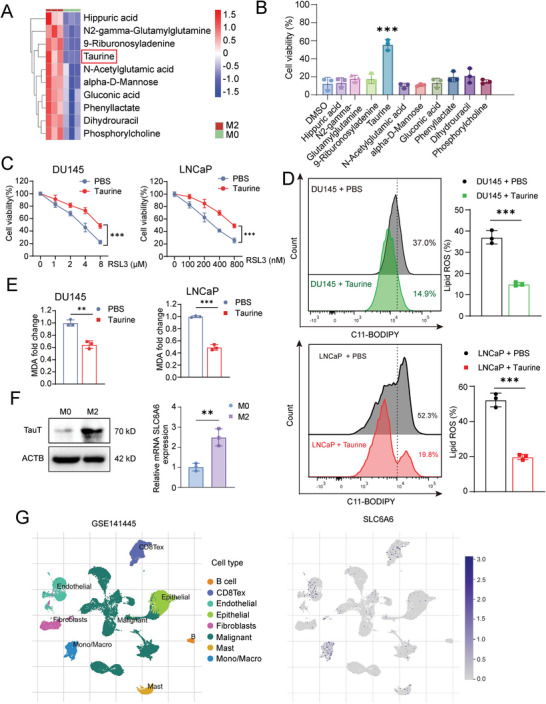
TAMs secrete taurine to suppress ferroptosis of PCa cells. A) Heatmap of top ten metabolites with significant differences in content between M0‐lCM and M2‐lCM using LC/MS assay. B) Cell viability of DU145 cells after treatment of ten metabolites for 24 h respectively following with the RSL3 treatment for another 24 h. The concentration used for each metabolite is listed in Table S2 (Supporting Information). C) Analysis of cell viability of PCa cells after incubated with taurine (100 µm) for 24 h following with the RSL3 treatment for another 24 h. D,E) Lipid peroxidation (D) and MDA level (E) of PCa cells after incubated with taurine (100 µm) for 24 h following with the RSL3 treatment for another 24 h. F) The mRNA and protein expression of TauT in M0 and M2 macrophage. G) Visualization of SLC6A6 gene expression in different cell types on a plot of scRNA‐seq profiles of human prostate cancer (GSE141445). Each experiment was performed in triplicate and independently repeated three times. (Two‐tailed Student's *t*‐test was used for the statistical analysis: ^**^, *p* < 0.01; ^***^, *p* < 0.001. Data are presented as means ± SD, *n* = 3).

In order to explore the specific low molecular weight components that suppress ferroptosis, M0‐lCM and M2‐lCM were processed for non‐target metabolomics analysis. According to the analysis, ten significantly enriched candidate metabolites were identified in M2‐lCM (Table S1, Supporting Information; **Figure** **2**A). Notably, we found that among these candidates examined, only M2 macrophage‐secreted taurine (Figure S2A–C, Supporting Information) showed an effective suppression on ferroptosis but not on other types of cell death, such as apoptosis, autophagy and necroptosis, according to cell viability (Figure 2B,C; Figure S2D–I, Supporting Information), lipid ROS as well as MDA assays (Figure 2D,E). Since the taurine transporter TauT, (also known SLC6A6 as its gene name) is a major export/import protein of taurine, we herein examined the expression of TauT in macrophages and found that TauT was indeed highly and specific expressed in M2 macrophages at both mRNA and protein levels (Figure 2F; Figure S3A, Supporting Information). Consistently, the analysis of published human PCa single‐cell RNA‐seq data also showed a major expression of TauT in epithelial tumor cells and tumor infiltratory macrophages (Figure 2G). As a confirmation, we collected both tumor and adjacent tissues from PCa patients (*n* = 6) and separated the tissues into two parts. One part was used to measure the content of taurine in tissues. The other part was used to sort macrophages and epithelial cells, and then to determine their expression of TauT respectively. A significantly higher level of taurine was observed in tumor tissues, compared to the adjacent tissues (Figure S3B, Supporting Information). As expected, the TauT expression was significantly upregulated in tumor‐infiltrated macrophages versus in macrophages in the adjacent normal tissues and in tumor cells versus in adjacent prostatic epithelial cells (Figure S3C, Supporting Information). However, in tumor tissues, no significant difference of either taurine content or the TauT expression was observed among tissues with different Gleason Scores (Figure S3D, Supporting Information). The TauT expressional data assay from the TCGA database also showed a similar result as we described above (Figure S3E, Supporting Information). On the other hand, when the taurine content and the TauT expression in tumor tissues with metastasis was compared to those without metastasis, no significant difference was observed either in these two groups (Figure S3F,G, Supporting Information). Moreover, we attempted to investigate the polarization state of macrophages in clinical samples, we found that the major tumor infiltrated macrophage was M2 macrophages (38.2%±4.6%) by flow cytometry assay (Figure S3H, Supporting Information), which was consistent with previous reports.^[^ ^21^, ^22^ ^]^ In addition, we confirmed that treatment with a high concentration of taurine showed no cytotoxicity to PCa cells in vitro (Figure S3I, Supporting Information). Thus, these above results indicated that M2 macrophage‐secreted taurine plays an important role in the inhibition of ferroptosis without a direct cytotoxicity in PCa.

### 2.3 Blockage of Taurine Export or Import Promotes Ferroptosis in PCa

**Figure 3:**
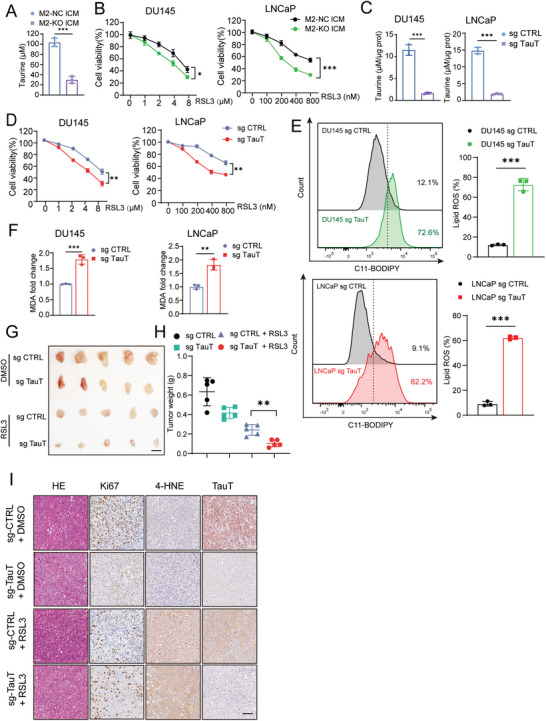
Blockage of taurine export/import by TauT knockout restores sensitivity of ferroptosis in PCa cells. A) Taurine level in M2‐NC lCM and M2‐KO lCM. B) Cell viability of PCa cells after incubated with M2‐NC lCM or M2‐KO lCM for 24 h following with the RSL3 treatment for another 24 h. C–F) Taurine level (C), cell viability (D), Lipid peroxidation level (E), and MDA level (F) in TauT knockout or control PCa cells with taurine (100 µm) treatment. G,H) Harvested xenografts (G) and tumor weight measurement (H) after inoculation of PCa cells with/without TauT knockout following with the RSL3 or DMSO treatment. (Scale bar = 1 cm, *n* = 5.) I) H&E and IHC staining in harvested xenografts (Scale bar = 100 µm). Each experiment was performed in triplicate and independently repeated three times. (Two‐tailed Student's *t*‐test was used for the statistical analysis: *, *p* < 0.05; **, *p* < 0.01; ***, *p* < 0.001. Data are presented as means ± SD, *n* = 3).

Next, considering as a potential therapeutic strategy, we wondered whether blocking taurine export in macrophage or blocking its import in PCa cells by repression of TauT expression could restore the sensitivity to ferroptosis in PCa cells. To this end, we knocked out TauT in M2 macrophages via CRISPR/Cas9 system (Figure S4A, Supporting Information). After TauT knock‐out, we indeed observed a decreased taurine level in the M2 macrophage culture supernatant (named M2‐KO lCM vs M2‐NC lCM as a control) (**Figure** **3**A). Pre‐treatment of PCa cells with the M2‐KO lCM resulted in a higher sensitivity to ferroptosis than M2‐NC lCM, which indicated that M2 macrophages‐secreted taurine was a major metabolite to repress ferroptosis in PCa (Figure 3B; Figure S4B, Supporting Information).

Consistently, after the endogenous TauT expression (Figure S4C, Supporting Information) was examined, we knocked out TauT in PCa cells to block its import, a significant decrease of intracellular taurine levels was observed (Figure 3C; Figure S4D,E, Supporting Information). Moreover, TauT knock‐out sensitized PCa cells to ferroptosis even when a high level of taurine (100 µm) is added in the culture medium, which was evidenced by a decreased survival rate, increased cell death, elevated lipid ROS and MDA levels in tumor cells (Figure 3D–F; Figure S4F,G, Supporting Information). To extend our work in an in vivo setting, TauT knockout DU145 cells or control cells were subcutaneously inoculated in nude mice. One week after inoculation, RSL3 was intratumorally injected into mice every two days for three weeks to induce ferroptosis. After the xenografts were harvested (Figure 3G), we measured tumor tissue weights (Figure 3H) and performed IHC staining (Figure 3I; Figure S4H, Supporting Information) in tissue sections. We found that RSL3 treatment in a combination with knock‐out of TauT showed the most beneficial ferroptosis‐mediated tumor killing effects, evidenced by low tumor weights, a downregulation of Ki67 expression (Figure S4I,J, Supporting Information), an upregulation of cell death (Figure S4K, Supporting Information) and the strong staining of 4‐HNE in related tissue samples (Figure S4L, Supporting Information). Therefore, our results indicated that intake of macrophage‐secreted taurine could effectively suppress ferroptosis in PCa. Blockage of taurine export or import by inhibition of TauT expression might be a potential strategy to restore the sensitivity to ferroptosis.

### 2.4 Taurine Protects PCa against Ferroptosis by Activating the LXRα/SCD1 Axis

**Figure 4:**
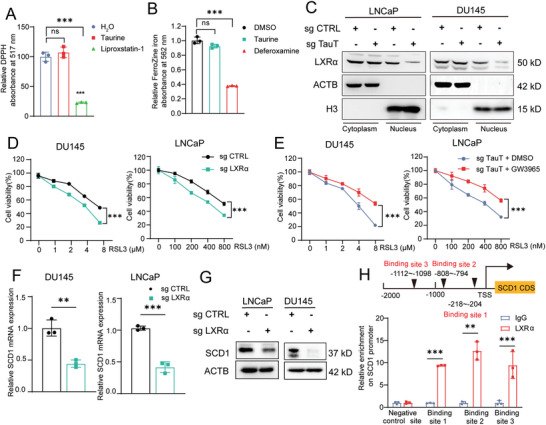
Taurine protects PCa against ferroptosis by activating the LXRα/SCD1 axis. A) The antioxidant activity of taurine was analyzed by a DPPH assay. Liproxstatin‐1 was used as a positive control. B) The iron chelator activity of taurine was analyzed by using the ferrozine Fe^2+^ binding assay. Deferoxamine (DFO) was used as a positive control. C) The LXRα expression in both cytoplasm and nuclear in PCa cells after TauT knockout. D) Cell viability analysis of PCa cells after LXRα knockout following with the RSL3 treatment for 24 h. E) Cell viability analysis of PCa cells after TauT knockout combined with GW3965 incubation following with the RSL3 treatment for 24 h. F,G) The mRNA (F) and protein expression (G) of SCD1 in PCa cells after LXRα knockout. H) Binding sites of LXRα transcription factors in the promoter region of SCD1. A black triangle indicates binding site(s) for LXRα transcription factors. Experiments presented in (C–H) were performed with the existence of taurine (100 µm). Each experiment was performed in triplicate and independently repeated three times. (Two‐tailed Student's *t*‐test was used for the statistical analysis: ns, not significant; ^**^, *p* < 0.01; ^***^, *p* < 0.001. Data are presented as means ± SD, *n* = 3).

In order to uncover the molecular mechanism of taurine‐induced anti‐ferroptosis, we first investigated whether taurine could exert its function through directly scavenging ROS or blocking process of ferroptosis in the manner of chelating iron ion. By diphenyl‐2‐picrylhydrazyl (DPPH) scavenging assay, we observed that taurine did not have the ability to directly scavenge ROS (**Figure** **4**A). Next, by ferrozine iron chelation assay, taurine also showed no chelation to iron compared to a positive control using deferoxamine (DFO) (Figure 4B). According to the above results, we speculated that similar to kynurenine as a ligand of Aryl Hydrocarbon Receptor (AHR), taurine might work as a ligand to activate a related downstream protein/receptor for anti‐ferroptosis.^[^ ^20^ ^]^ Actually, it was reported that liver X receptor‐alpha (LXRα) was able to interact with taurine during the process of lipogenesis.^[^ ^23^ ^]^ Meanwhile, LXRα was also reported to be involved in the promotion of chemoresistance in triple negative breast cancers.^[^ ^24^ ^]^ Combined our observation with these previous finding, we herein hypothesized that taurine might activate LXRα to promote the expression of its downstream target genes to inhibit ferroptosis. Since nuclear translocation of LXRα was reported as an activated status of LXRα,^[^ ^25^ ^]^ to test our hypothesis, we examined the intracellular localization of LXRα with or without the existence of taurine to check its activity. We found that after knock out of TauT to disrupt the import of taurine in PCa, LXRα was mostly sequestrated in the cytoplasm with limited location in the nucleus, which indicated that taurine might be a major ligand to activate LXRα and to promote its nuclear translocation (Figure 4C; Figure S5A, Supporting Information). Functionally, direct knock‐out of LXRα restored the sensitivity to ferroptosis in PCa even with the treatment of taurine (Figure 4D; Figure S5B,C, Supporting Information). Similarly, directly activated LXRα using its agonist GW3965 inhibited ferroptosis even under the condition of TauT knock‐out in PCa (Figure 4E). So, these evidence supported our speculation that intake of taurine in PCa cells could inhibit ferroptosis via activation of LXRα.

**Figure 5:**
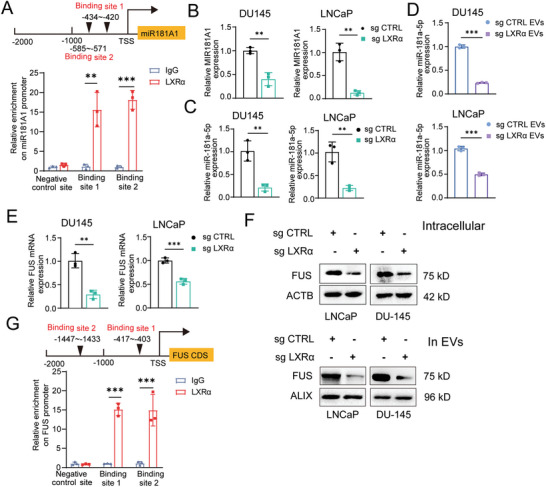
LXRα activates the transcription of MIR181A1 and FUS. A) Binding sites prediction and ChIP assay confirmation of LXRα transcription factors in the promoter region of the host gene of miR181A1. B,C) The intracellular expression of precursors miR181A1 (B), and mature miR‐181a‐5p (C) in PCa cells after LXRα knockout. D) The level of miR‐181a‐5p in EVs derived from PCa cells in which LXRα was knock‐outed. E,F) The mRNA (E) and protein levels (F) of FUS in both PCa cells and in tumor cell‐derived EVs after LXRα knockout. G) Binding sites prediction and ChIP assay confirmation of LXRα transcription factors in the promoter region of FUS. All of the experiments were performed with the treatment of taurine (100 µm). Each experiment was performed in triplicate and independently repeated three times. (Two‐tailed Student's *t*‐test was used for the statistical analysis: ^**^, *p* < 0.01; ^***^, *p* < 0.001. Data are presented as means ± SD, *n* = 3).

Next, we further investigated potential target genes of LXRα for inhibition of ferroptosis in PCa. Since stearoyl‐CoA desaturase 1 (SCD1) was a well reported gene to be tightly related to inhibition of ferroptosis^[^ ^11^ ^]^ and was indicated as a target gene of LXRα in macrophages,^[^ ^23^ ^]^ we herein first investigated whether the expression of SCD1 could be promoted by LXRα in PCa. For this purpose, we first analyzed the endogenous SCD1 expression (Figure S5D, Supporting Information) and then knocked‐out LXRα expression in DU145 cells to evaluate the change of SCD1 expression. As a result, a decreased expression of SCD1 was observed at both mRNA and protein levels (Figure 4F,G). Bioinformatic assay along with ChIP assay also confirmed that SCD1 was a direct target gene of LXRα in PCa (Figure 4H), which indicated that activated LXRα could suppress ferroptosis by enhancing the expression of SCD1. Besides coding genes such as SCD1, we wondered whether there are non‐coding genes (e.g., microRNAs and lncRNAs) also regulated by LXRα as its target genes during the process of inhibition of ferroptosis (Figure S6A, Supporting Information). We performed target prediction assay and found that miR‐181a‐5p was a potential target of LXRα (Figure S6B, Supporting Information), which was reported to be enriched in serum EVs from bone‐metastatic PCa patients in our previous work.^[^ ^26^ ^]^ As shown in **Figure** **5**A, two binding sites of LXRα were found to be located in the promoter of the host gene of miR‐181a‐5p precursor MIR‐181A1 by ChIP assay. We found that treatment with taurine in DU145 and LNCaP cells upregulated the expression of both miR‐181a‐5p and its precursor MIR‐181A1 (Figure S6C,D, Supporting Information). In contrast, inhibition of LXRα or TauT expression significantly downregulated the intracellular expression of both miR‐181a‐5p precursor and mature miR‐181a‐5p (Figure 5B; Figures S5C and S6E,F, Supporting Information). Additionally, we studied the enrichment of miR‐181a‐5p in EVs derived from DU145 cells with or without LXRα knock‐out. Consistent with the intracellular expression of miR‐181a‐5p, inhibition of LXRα attenuated the enrichment of miR‐181a‐5p in EVs (Figure 5D). Given the fact that FUS was an RNA‐binding protein for recruitment of miR‐181a‐5p into EVs,^[^ ^27^ ^]^ we wondered whether LXRα knock‐out also downregulated the expression of FUS so to interfere the EV enrichment of miR‐181a‐5p. As expected, we observed a decrease of FUS level in both tumor cells and tumor‐derived EVs after LXRα knock‐out (Figure 5E,F). Furthermore, by performing bioinformatic assay in a combination with ChIP assays, we confirmed that there were two predicted binding sites between LXRα and the promoter region of FUS, which supported idea that FUS is a downstream target gene of LXRα (Figure 5G). Taken together, our findings indicated that taurine could protect PCa against ferroptosis by activating the LXRα/SCD1 axis. The latter promoted the expression of miR‐181a‐5p and the expression of FUS as well for enrichment of miR‐181a‐5p into tumor derived EVs.

### 2.5 MiR‐181a‐5p Enriched EVs Promoted M2 Polarization of Macrophages to Suppress Ferroptosis in PCa Cells

**Figure 6:**
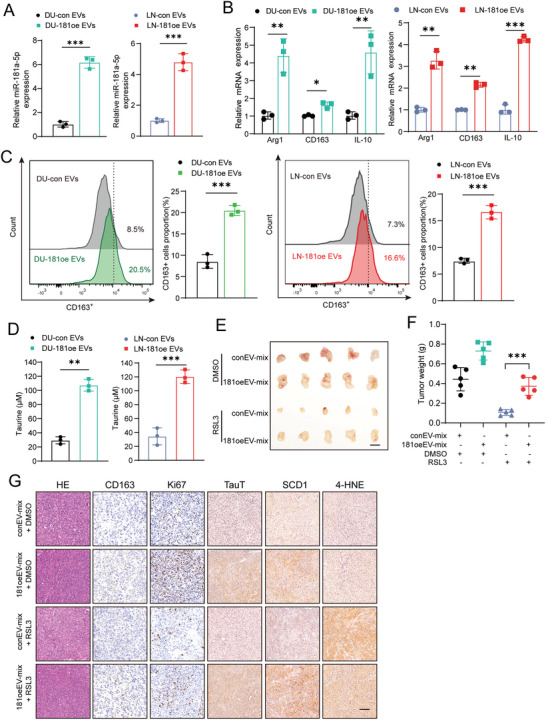
MiR‐181a‐5p‐enriched EVs promote M2 polarization of macrophages to suppress ferroptosis in PCa cells. A) The expression of mature miR‐181a‐5p in macrophages after incubated with relevant EVs. B) The expression of M2 macrophage markers CD163, Arg‐1, and IL10 in macrophages after incubated with relevant EVs. C) Flow cytometry was performed to analyze the effect of PCa cell‐derived miR‐181a‐5p‐enriched EVs on promotion of the expression of the typical M2 marker CD163. D) Taurine level in macrophages after incubated with relevant EVs. E,F) Harvested xenografts (E) and tumor weight measurement (F) after inoculation of a mixture of related macrophages‐PCa cells following treatments with RSL3 or DMSO. (Scale bar = 1 cm). G) H&E and IHC staining in harvested xenografts (Scale bar = 100 µm). Each experiment was performed in triplicate and independently repeated three times. (Two‐tailed Student's *t*‐test was used for the statistical analysis: *, *p* < 0.05; **, *p* < 0.01; ***, *p* < 0.001. Data are presented as means ± SD, *n* = 3).

Since tumor‐derived EV was well reported to transport microRNAs as inducers to promote polarization of macrophage for tumor progression,^[^ ^17^ ^]^ we herein further investigated whether miR‐181a‐5p‐enriched EVs could be taken in by macrophages to promote their polarization and even to facilitate taurine production. To this end, we stably overexpressed miR‐181a‐5p in LNCaP and DU145 cells (Figure S7A,B, Supporting Information) and then harvested relevant EVs (named LN‐181oe EVs vs LN‐con EVs as a control and DU‐181oe EVs vs DU‐con EVs as a control, respectively). By nanoparticle tracking analysis (NTA), transmission electron microscopy (TEM) assay and western blot assay, we confirmed similar characteristics of EVs no matter whether miR‐181a‐5p was overexpressed or not (Figure S7C–E, Supporting Information). Also, the intake of miR‐181a‐5p‐enriched EVs was not significantly different compared to that of the control ones (Figure S7F,G, Supporting Information). Next, we investigated whether endogenous expression of miR‐181a‐5p and its precursor was changed during the process of M1 and/or M2 polarization. We induced M1 or M2 polarization by using LPS + INF‐g or IL‐4 + IL‐13 respectively and no significant change of either miR‐181a‐5p or its precursor was observed, indicating that the process of polarization had almost no effect on the endogenous expression of miR‐181a‐5p (Figure S7H,I, Supporting Information). Based on this observation, we incubated M0 macrophages with miR‐181a‐5p‐enriched EVs and found that the intracellular content of mature miR‐181a‐5p but not its precursor was increased along with the upregulation of the expression of M2 macrophage marker gene, indicating the increased M2 polarization (**Figure** **6**A–C; Figure S7J, Supporting Information). Moreover, we found that incubation of M0 macrophages with miR‐181a‐5p‐enriched EVs promoted their secretion of taurine (Figure 6D). For an in vivo assay, we first incubated M0 macrophages with DU‐181oe EVs or DU‐con EVs, then these pre‐incubated macrophages were mixed with DU145 cells (1: 4) to be co‐inoculated subcutaneously in nude mice following with the treatment of RSL3 (Figure S8A, Supporting Information). By measurement of xenografts’ weights, we found that the tumor growth was enhanced in the DU‐181oe EVs incubation group compared to the control group, indicating an effective inhibition of ferroptosis in PCa (Figure 6E,F). By IHC assay, we also observed that the expression of SCD and Ki67 was upregulated (Figure S8B, Supporting Information), the content of 4‐HNE and cell apoptosis (Figure S8C,D, Supporting Information) was downregulated after treatment with RSL3 in DU‐181oeEVs incubation group versus control group, as evidence for ferroptosis resistance (Figure 6G). On the other hand, we also directly overexpressed miR‐181a‐5p in M0 macrophages as an efficacy control in order to evaluate whether intake of miR‐181a‐5p was a major cause of M2 polarization and enhancement of taurine secretion in macrophages (Figure S8E, Supporting Information). Consistent with the results with incubation with miR‐181a‐5p‐enriched EVs, direct overexpression of miR‐181a‐5p could effectively promote M2 polarization and taurine secretion (Figure S8F–H, Supporting Information). Taken together, these data indicated that intake of miR‐181a‐5p in macrophage promoted its M2 polarization, and in turn increased taurine secretion to protect PCa from ferroptosis.

### 2.6 MiR‐181a‐5p Promoted M2 Macrophage Polarization and Upregulated TauT Expression via Targeting the Hippo Signaling Pathway

**Figure 7:**
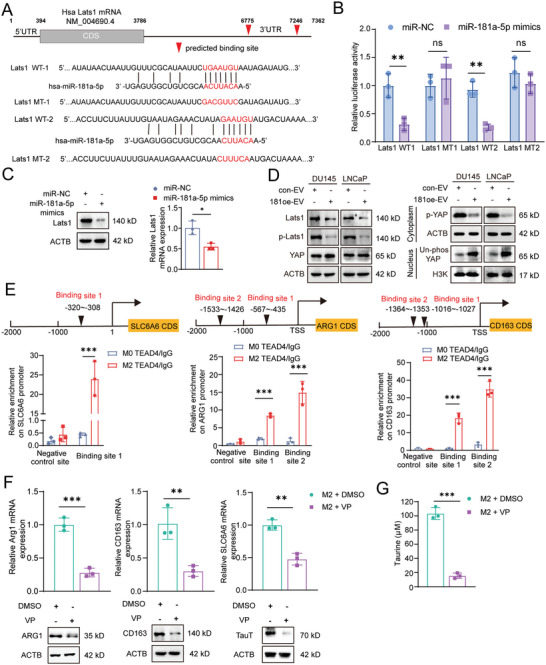
miR‐181a‐5p promotes M2 macrophage polarization and upregulates TauT expression via inhibiting the hippo signaling pathway. A) Wild‐type (WT) or mutant (MT) sequence of predicted binding sites of miR‐181a‐5p on 3′‐UTR of Lats1 mRNA. B) luciferase reporter assay for confirmation the binding of miR‐181a‐5p on its binding sites in 3′‐UTR of Lats1 mRNA. C) The expression of LATS1 on mRNA and protein levels in M0 macrophages after overexpression of miR‐181a‐5p mimics. D) The expression of Hippo pathway and its downstream YAP in M0 macrophage after incubation with relevant EVs. E) Binding sites prediction and ChIP assay of TEAD4 transcription factor in the promoter regions of ARG1, CD163, and SLC6A6. F) The mRNA and protein expression of ARG1, CD163, and TauT in M2 macrophages after treatment with verteporfin (VP) versus DMSO. G) Taurine level in M2 macrophages after treatment with verteporfin (VP) versus DMSO. Each experiment was performed in triplicate and independently repeated three times. (Two‐tailed Student's *t*‐test was used for the statistical analysis: ns, not significant; *, *p* < 0.05; **, *p* < 0.01; ***, *p* < 0.001. Data are presented as means ± SD, *n* = 3).

To explore the putative target of miR‐181a‐5p, we performed bioinformatic assay to search and identify the possible target gene. Given that it has been reported that under certain conditions, inhibition of the hippo pathway could induce either M1 polarization or M2 polarization,^[^ ^28^, ^29^ ^]^ we focused on whether and how miR‐181a‐5p regulated M2 polarization via the hippo‐YAP pathway. Notably, we found that Lats1, which is a well‐reported key negative regulator,^[^ ^30^ ^]^ was one of the putative miR‐181a‐5p target genes based on our data‐mining using the miRDB online software. Therefore, we then investigated whether promotion of M2 polarization due to intake of miR‐181a‐5p was achieved via targeting Lats1. First, by bioinformatic analysis and luciferase reporter assay, we found two binding sites of miR‐181a‐5p in the 3′UTR of with lats1 mRNA (**Figure** **7**A,B). Then, Overexpression of miR‐181a‐5p in M0 macrophages downregulated the expression of lats1 (Figure 7C). Moreover, either incubation with miR‐181a‐5p‐enriched EV or overexpression of miR‐181a‐5p inhibited the Hippo pathway via targeting lats1, which resulted in an increased nuclear translocation of YAP1 (Figure 7D; Figure S9A, Supporting Information). In a rescue assay, this increased translocation could be reversed after overexpression of vectors containing lats1 CDS (Figure S9B, Supporting Information). At the same time, a decreased CD163‐positive population of M2 macrophages was observed after restoration of lats1 expression, which indicated that miR‐181a‐5p promoted M2 macrophage polarization via inhibition of the Hippo pathway (Figure S9C,D, Supporting Information).

Given the fact that nuclear located YAP could work as a co‐activator of the transcriptional factor TEAD family (including TEAD1‐4) to promote the transcription of their target genes, we herein performed ChIP assay to check whether M2 macrophage markers and TauT were potential target genes of YAP‐TEAD complex. As shown in Figure 7E, there was one binding site of TEAD4 in the promoter region of TauT and two binding sites of TEAD4 in the promoter region of ARG1 and CD163, respectively. Therefore, these data suggested that TauT, ARG1, and CD163 are likely target genes of the YAP‐TEAD complex. To provide further evidence for this notion, we treated the microphage cultures with a YAP inhibitor Verteporfin (VP)^[^ ^31^ ^]^ after either incubation with miR‐181a‐5p‐enriched EV or overexpression of miR‐181a‐5p, repressed expression of ARG1, CD163, and TauT was observed along with the attenuated secretion of taurine (Figure 7F,G). Collectively, these data demonstrated that intake of miR‐181a‐5p from tumor‐derived EVs inhibited the Hippo pathway in macrophages to promote its M2 polarization as well as the secretion of taurine, serving for resistance to ferroptosis in PCa.

## 3 Discussion

**Figure 8:**
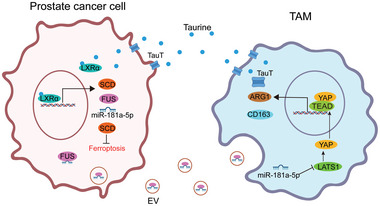
The crosstalk between TAMs and PCa cells promotes the resistance of ferroptosis in PCa cells. Tumor derived miR‐181a‐5p enriched EVs promote M2 macrophage polarization via targeting the hippo pathway to upregulate the expression of TauT and the secretion of taurine so to enhance the resistance of ferroptosis in PCa as a positive feedback loop.

Induction of ferroptosis is a prospective strategy for treatment‐resistant of tumors including prostate cancer.^[^ ^32^ ^]^ Emerging evidence has suggested that factors in microenvironment, such as metabolites and EVs, can activate antioxidant mechanisms and suppress ferroptosis in tumor.^[^ ^16^, ^33^ ^]^ In this study, we found that taurine, a metabolite of TAM, could effectively inhibit ferroptosis in PCa by activating the LXRα/SCD1 aixs. As a potential intervention, besides direct knockdown of LXRα, blocking taurine import via inhibition of TauT expression restored the sensitivity of ferroptosis in PCa. On the other hand, we found that LXRα could also activate the transcription of miR‐181a‐5p and FUS, resulting in an enrichment of miR‐181a‐5p into tumor‐derived EVs. These miR‐181a‐5p‐enriched EVs was taken in by macrophages and induced their polarization to adapt the M2 phenotype via inhibition of lats1 expression to repress the Hippo pathway, resulting in the nuclear translocation of YAP to upregulate the expression of M2 macrophage marker genes such as ARG1 and CD163 as well as TauT. Our findings demonstrated that the macrophage secreted‐taurine could induce anti‐ferroptosis in PCa. Also, our data indicated a shuttle of miR‐181a‐5p from tumor cells to macrophages for the enhancement of taurine secretion as a feedback response so to form a macrophage‐tumor cell reciprocal communication (**Figure** **8**).

Although taurine has been shown to be able to directly react with arachidonic acid to inhibit ferroptosis in lung cancer,^[^ ^34^ ^]^ our current study identified a different protective mechanism for the resistance to ferroptosis. We found that taurine can activate the LXRα receptor and its downstream gene SCD1 to repress ferroptosis in PCa. There are serval lines of evidence to support our model. First, support for our study comes from previous reports, in which taurine is shown to be capable to bind to LXRα during the process of lipogenesis in hepatocytes^[^ ^20^ ^]^ and LXRα activates the transcription of SCD1 in human preadipocytes.^[^ ^35^ ^]^ Rather than in lipogenesis, our study reveals that taurine can activate LXRα and upregulate the expression of SCD1 during the resistance to ferroptosis in PCa. Secondly, we observed that tumor‐infiltrated macrophages appeared to be a major source of taurine in the tumor microenvironment besides to a direct uptake from food. Thirdly, as a major transporter for taurine export and import,^[^ ^36^ ^]^ the expression of TauT was observed to be upregulated in both M2 macrophages and PCa cells, although at the same time, we also observed TauT expression in other cell types including CD8+ T cells.^[^ ^37^ ^]^ Therefore, our findings indicate that during the resistance to ferroptosis, the crosstalk between tumor cells and macrophages is established mainly due to the high TauT expression level in both two types of cells for transport of taurine. On the other hand, we cannot rule out the possibility that besides macrophages, T cells, endothelial cells and fibroblasts might also be potential taurine‐secretion cells. So detailed exploration of the TauT function and the regulation of taurine secretion in other intrinsic cells such as T cells are need to be carried out. Moreover, inhibition of TauT expression could attenuate the efficacy of anti‐ferroptosis in PCa. Thus, our results suggested that attenuating TauT‐mediated taurine export/import might be a promising adjuvant therapeutic strategy to restore the sensitivity to ferroptosis during PCa treatment.

It is believed that the crosstalk between tumor cells and macrophages play an important role during the process of therapeutic resistance including anti‐ferroptosis in tumors.^[^ ^38^ ^]^ However, the detailed mechanism is so far still not understood. It is worth mentioning that besides an IL‐6/JAK2/STAT3 axis‐dependent induction of a macrophage‐tumor cell crosstalk as described previously,^[^ ^9^ ^]^ our study uncovered an EV‐mediated crosstalk between tumor cells and macrophages. That is, tumor‐derived EVs shuttle miR‐181a‐5p from tumor cells to macrophages to promote the M2 polarization for anti‐ferroptosis in PCa. In detail, after intake of miR‐181a‐5p in macrophages, it inhibits the expression of Lats1 to inactivate the Hippo pathway to promote M2 polarization and in turn to increase taurine secretion. We have following data to support our novel mechanism of the crosstalk. First, besides promoting the expression of SCD1 which is the downstream effector for anti‐ferroptosis, LXRα also upregulated the levels of miR‐181a‐5p and its RNA‐binding protein FUS,^[^ ^27^ ^]^ thereby enhancing the enrichment of miR‐181a‐5p in tumor‐released EVs. Secondly, our study uncovers that macrophages are likely recipient cells of the miR‐181a‐5p‐enriched EVs. Intake of miR‐181a‐5p in microphages downregulates the expression of Lats1, leading to inactivation of the Hippo pathway and in turn the increase of nuclear translocation of YAP. Thirdly, the nuclear translocated YAP upregulates the expression of M2 polarization‐related genes ARG1 and CD163, as well as taurine transport gene TauT so that both the M2 polarization of macrophages and the export of taurine are enhanced. Finally, as a feedback messenger, macrophage‐secreted taurine helps tumor cells to resist ferroptosis, which constitutes the reciprocal crosstalk between tumor cells and microphages.

In summary, in this study we demonstrate that TAMs export taurine to inhibit ferroptosis by activation of the LXRα/SCD1 axis in PCa. Meanwhile, LXRα promotes the expression and recruitment of miR‐181a‐5p into tumor cell‐released EVs. As a response, EVs‐mediated shuttle of miR‐181a‐5p into macrophages induces M2 polarization of macrophages via inhibition of the Hippo pathway to enhance the export of taurine as a positive feedback loop. Based on our findings, disruption of taurine transport through blockage of taurine import/export gene TauT using relevant antibodies or small molecular inhibitors might be a potential adjuvant treatment to attenuate this macrophage‐tumor cell crosstalk to restore the sensitivity to ferroptosis in PCa in clinics.

## 4 Experimental Section

### Cell Culture

The human PCa cell lines LNCaP, DU‐145, PC‐3, CWR22‐Rv1, and the human monocytic leukemia cell line THP‐1 were obtained from the Type Culture Collection of the Chinese Academy of Sciences (Shanghai, China). These cells were cultured in Dulbecco's modified Eagle's medium (DMEM, for LNCaP and DU‐145) (Gibco, C11995500BT) or RPMI 1640 (for THP‐1) (Gibco, C11875500BT) supplemented with 10% fetal bovine serum (Gibco, 10270‐106) and 100 U mL^−1^ penicillin/streptomycin (Beyotime, ST488) at 37 °C with 5% CO_2_ and authenticated by short tandem repeat (STR) profile analysis.

Bone marrow‐derived macrophages (BMDM) were obtained by flushing cells from the femurs and tibias of mice. Cells were cultured with RPMI‐1640 containing 10% FBS and 20 ng ml^−1^ M‐CSF (Peprotech,315‐02) for 7 days (replace medium at day3) to induce differentiation. The differentiated cells (i.e., BMDM M0 macrophages) were then treated with 100 ng ml^−1^ LPS (Biosharp, BS904) and 20 ng ml^−1^ IFN‐γ (Peprotech, 315‐05) for polarization to M1 macrophages or 20 ng ml^−1^ IL‐4 (Peprotech, 214‐14) combined with 10 ng ml^−1^ IL‐13 (Peprotech, 210–13) for polarization to M2 macrophages, respectively.

### Macrophage Polarization and Coculture Experiment

THP‐1 were differentiated into M0 macrophages by treatment with 100 ng ml^−1^ phorbol 12‐myristate 13‐acetate (PMA) (sigma, P1585) for 24 h. M0 macrophages were rested for 24 h and then were added 20 ng ml^−1^ IL‐4 (Peprotech, 200–04) combined with 10 ng ml^−1^ IL‐13 (Peprotech, 200–13) for M2 polarization or 100 ng ml^−1^ LPS (Biosharp, BS904) combined with 20 ng ml^−1^ IFN‐γ (Peprotech, 300–02) for M1 polarization, respectively. For coculture experiment, macrophages were seeded in the upper chamber of a 0.4 µm pore transwell insert (Corning, 3412) and PCa cells were cultured in the lower chamber.

### Conditioned Medium

After macrophages were incubated with EVs or treated with IL4 and IL‐13 for 24 h, the medium was replaced with fresh basic RIPM 1640 to culture for another 24 h. The supernatant was collected, centrifuged at 300 g for 10 min to remove cell debris, filtered through a 0.22 µm pore filter (Corning, 430517) and the flow through medium was named conditioned medium (CM). To separate high molecular weight components, CM was centrifuged with an Amicon Ultra‐15 centrifugal filter unit (10 kDa, Millipore, UFC9010) at 4000 g for 60 min. The upper concentrated medium was diluted with basic RIPM 1640 and named high molecular weight conditioned medium (hCM). The flow through medium was named low molecular weight conditioned medium (lCM).

### Clinical Samples

The investigation was conducted in accordance with the ethical standards and according to the Declaration of Helsinki (World Medical Association Declaration of Helsinki. Ethical Principles for Medical Research Involving Human Subjects, 1964), national and international guidelines as well as the Committee for Ethical Review of Research Involving Human Subjects at Ren Ji Hospital (approval number: RH‐2021‐132). Prostate cancer patients' tumor tissue was obtained from the Department of Urology, Renji Hospital of Shanghai Jiaotong University Medical School. Six tumor tissues and paired tumor‐adjacent tissue from PCa patients were separated into two parts. One part was used for sorting of macrophages (CD45^+^CD11b^+^CD68^+^) and epithelial cells (CD45^−^EPCAM+) by flow cytometry and in turn to determine mRNA expression of SLC6A6 by qRT‐PCR. Another part was used to assess the levels of taurine. Another three prostatic tumor tissue was employed to analyze the polarization state of macrophage by flow cytometry. All specimens were obtained with the informed consent of the patients. Details of the antibodies used are provided in Table S3 (Supporting Information). The clinical information of prostate cancer patients was showed in Table S4 (Supporting Information).

### EV Purification and Identification

DMEM or RIPM1640 medium containing 10% EV‐depleted FBS (Gibco, A2720801) was used for cell culture before the isolation of EVs. EVs were isolated from the culture medium by differential centrifugation as previously reported.^[^ ^39^ ^]^ Briefly, cell debris was removed after centrifuging culture medium in turn at 300 g for 10 min, 2000 g for 10 min, and 10 000 g for 30 min. Then the cell supernatant was centrifuged at 110 000 g for 70 min (all steps were performed at 4 °C). EVs were collected and resuspended in PBS. EVs were subjected to transmission electron microscopy (TEM) and nanoparticle tracking analysis (NTA) to analyze the morphology and size distribution. Western blot assay was used to check the expression of EV biomarkers.

### Taurine Assay

To determine the taurine content, a taurine assay kits was used (Cell Biolabs, MET‐5071). Pretreated cells or patient‐derived tissue was homogenized in cold PBS, and protein content was determined using BCA assay. The homogenate was then mixed with taurine reaction buffer and incubated at room temperature for 30 min. The reaction was stopped by adding a stop solution, and the absorbance of each sample was measured at 405 nm using a microplate reader. The concentration of taurine in the samples was calculated using a standard curve generated from the absorbance values of the taurine standards provided in the kit.

### Cell Transfection

Macrophages were transfected with miR‐181a‐5p mimics (Ribobio, miR10000256‐1‐5) or miR‐NC mimics (Ribobio, miR1N0000001‐1‐10) using riboFECT CP transfection kit (Ribobio, C10502‐05) at a final concentration of 100 nm. The LATS1 overexpression plasmids were purchased from Miaoling biology (P22816). LXRα−3xFlag was cloned into pLenti‐CMV‐MCS‐T2A‐Puro vector (Miaolingbio, 73582) plasmid backbone to overexpress LXRα. Dual sgRNA CRISPR/Cas9 system (Addgene, 52961) mediated gene deletion of SLC6A6 and LXRα were constructed. The sgRNA sequences were as follows: SLC6A6 sgRNA1: 5′‐GACATCCTGAAGCCCTCACCA‐3′, SLC6A6 sgRNA2: 5′‐GCTAGACCACTTCTCCCTCTG‐3′. LXRα sgRNA1: GCAGCAGCTGCATCCTCAGAG, LXRα sg RNA2: GCCCACAGCCCTGCTCACCA.

### *mRNA* Extraction and qRT‐PCR Analysis

Total RNAs were extracted using an RNAfast200 kit (Fastagen, 220011). EV‐containing microRNAs were extracted using exoRNeasy Midi Kit (Qiagen, 77144). MicroRNA reverse transcription and qRT‐PCR analysis were performed using Taqman miRNA reverse transcription kit (Applied Biosystems, 4366596) and Taqman premix (Takara, RR390A), respectively. Total cDNA was synthesized using HiScript II Reverse Transcriptase (Vazyme, R201‐02) and then amplified using ChamQ Universal SYBR qPCR Master Mix (Vazyme, Q711‐02). The corresponding primers and probe sequences were listed in Table S5 (Supporting Information).

### Chromatin Immunoprecipitation (ChIP) Assay

The ChIP assay was performed using the SimpleChIP Plus Enzymatic Chromatin IP Kit (Magnetic Beads) (CST, 9003S). Briefly, 1 × 10^7^ DU145 cells overexpressed with LXRα or 1 × 10^7^ M0/M2 macrophages were fixed with 1% formaldehyde and sonicated for 60 s. Tead4 antibody, Flag antibody or normal rabbit IgG was incubated with the solubilized chromatin overnight at 4 °C, respectively. Pull‐down ChIP DNA was quantified by real‐time PCR. Putative LXRα or TEAD4 binding chromatin regions were predicted by using the Contra V3 online software (http://bioit2.irc.ugent.be/contra/v3) or JASPR database (https://jaspar.genereg.net/), respectively. Details of the antibodies used are provided in Table S3 (Supporting Information). The corresponding ChIP primers are listed in Table S6 (Supporting Information).

### Luciferase Reporter Assay

For the dual luciferase assay, wild type (WT) or mutant (MT) putative miR‐181a‐5p binding site sequences on lats1 mRNA 3′‐UTR region were purchased from Genomeditech. Next, THP1‐derived M0 macrophages (1 × 10^6^ cells) were co‐transfected with 0.5 µg above WT or MT binding site sequences containing luciferase reporter plasmids, miR‐181a‐5p or NC mimics and 0.05 µg Renilla luciferase vector using Lipofectamine 3000 (Invitrogen, L3000015). Fourty‐eight hours after transfection, luciferase activity was measured using the dual luciferase reporter assay system (Promega, E1910). Relative luciferase activity was obtained by normalization of firefly luciferase activity to the Renilla luciferase activity.

### Cytoplasmic and Nuclear Protein Extraction and Western Blot

For total protein harvest, cells were harvested and lysed on ice using cell lysate (Beyotime, P0013) containing protease inhibitors cocktail (MCE, HY‐K0011). In order to extract cytoplasmic and nuclear proteins respectively, the Minute Cytoplasmic and Nuclear Extraction Kit (Sangon Biotech, C510001) was used. All of the lysates were boiled for 10 min to denature and inactivate the protein samples for western blot assay.

Briefly, proteins were separated by SDS‐PAGE and transferred to polyvinylidene fluoride membrane (Millipore, IPVH00010). The membranes were blocked in 5% non‐fat milk for 1 h at room temperature and then incubated with indicated diluted primary antibody overnight at 4 °C. Membranes were then incubated with the secondary antibodies for 1 h at room temperature. Chemiluminescence reagents (Millipore, P90720) were used to capture protein bands in the Molecular Imager System (BIO‐RAD). Details of the antibodies used are provided in Table S3 (Supporting Information).

### DPPH Scavenging Assay

2,2‐Diphenyl‐1‐picrylhydrazyl (DPPH) (MCE, HY‐112053) was dissolved in methanol at 200 µm as a dilution. Taurine (Sigma, T8691) or Liprostatin‐1 (MCE, HY‐12726) was mixed with DPPH dilution respectively to a final concentration at 200 µm. Liprostatin‐1 was used as a positive control. After incubation for 10 min, the absorbance was measured at 517 nm using a BioTek Synergy HT microplate reader (BioTek). Results were normalized to the H_2_O control.

### Ferrozine Iron Chelation Assay

The concentration of Iron (II) chloride (Sangon Biotech, A501386) in water was diluted to 10 µm. Subsequently, the tested compounds were added to 1 ml of the solution containing 40 µm Iron (II) chloride and incubated for 10 min at room temperature. Then, 3‐(2‐pyridyl)−5,6‐diphenyl‐1,2,4‐triazine‐p, p′‐disulfonic acid monosodium salt hydrate (FerroZine) (MCE, HY‐137805) was added and mixed to achieve a final concentration of 20 µm. The samples were thoroughly mixed and incubated at room temperature for 1 h. Deferoxamine (DFO) (MCE, HY‐B0988) was used as a positive control. The absorbance was measured at 562 nm using a microplate reader. The iron‐chelating activity of the tested compounds was normalized to that of DMSO, which has no iron‐chelating activity and was set as 100%.

### Measurement of Lipid Peroxidation

The intracellular Malondialdehyde (MDA) level was measured with a Lipid Peroxidation MDA Assay Kit (Beyotime, S0131S) according to the manufacturer's instructions. The value was normalized to the protein concentration detected by a Pierce BCA protein assay kit (Thermo Scientific, 23227). Lipid ROS was detected using a BODIPY 581/591 C11 probe (Thermo Scientific, D3861). Briefly, cells were incubated with 2 µm BODIPY 581/591 C11 at 37 °C for 30 min and then the fluorescence was detected on a flow cytometer at fluorescein isothiocyanate (FITC) green channel (LSR Fortessa; BD). A minimum of 10 000 live cells were collected and data were then analyzed by FlowJo software.

### Immunohistochemistry (IHC) and Immunofluorescent (IF) Staining

Tissue samples were fixed in 10% formaldehyde, embedded in paraffin, and sectioned (4 µm in thickness) for IHC or IF staining. Sections were deparaffinized and rehydrated, followed by inactivation of endogenous peroxidase activity with 3% H_2_O_2_(Aladdin, H112517) in methanol, blocked with 5% donkey serum (Solarbio, SL050). The sections were then incubated with the relevant primary antibodies overnight at 4 °C. The samples were washed by PBS and incubated with peroxidase‐labeled secondary antibodies for 1 hour, followed by diaminobenzidine (DAB) staining (Vector Laboratories, SK‐4100).

PCa cells transfected with sg CTRL or sg TauT were plated in glass coverslips in a 24‐well culture plate. The adherent cells were fixed with 4% paraformaldehyde for 10 min at room temperature, permeabilized with 0.05% Triton‐100 for 10 min, blocked with 5% donkey serum for 1 h at room temperature. Next, the specimens were incubated with anti‐LXRα antibody at the dilution of 1:100 overnight. Then the specimens were washed with PBS and stained with Alexa Flour 594‐labeled second antibody at room temperature for 2 h. Specimens were counterstained with DAPI (Sigma, D9542) and mounted with DAPI‐containing media (Biosharp, BL739A). All images were captured with a microscope (Leica, DFC420C).

### Fluorescence Microscopy

M0 macrophages (1 × 10^7^) were seeded on coverslips in a 24‐well plate and cocultured with PKH67 (Sigma, PKH67GL)‐labeled EVs or EVs containing Cy3 labeled miR‐181a‐5p (miR‐181a‐5p‐Cy3) at 37 °C for 4 h.^[^ ^40^ ^]^ Specimens were counterstained with DAPI (Sigma, D9542) and mounted with DAPI containing media (Biosharp, BL739A). All images were captured with a microscope (Leica, DFC420C).

### Non‐Targeted Metabolomics Analysis

The culture medium of M0 or M2 macrophages was refreshed with basic medium to culture for another 24 h. After that, the supernatant was in turn collected into 50 ml centrifugal tube, centrifuged at 300 g for 10 min, filtered using Amicon Ultra‐15 centrifugal filter units for preparing low molecular weight condition medium (lCM). The lCM samples were frozen with liquid nitrogen for the subsequent analysis. The samples were subjected to Metabolomics Platform (BioNovoGene, Suzhou, China) for non‐targeted metabolomics analysis. Top 10 differential metabolites in M2‐lCM versus M0‐lCM identified by non‐target metabolomics analysis were listed in Table S1 (Supporting Information).

### CCK8 Cell Viability Assay

A Cell Couting Kit‐8 (CCK8) (Biosharp, BS350A) was used to determine cell viability. Cells (1 × 10^4^) were cultured in a 96‐well plate and were pretreated with relevant conditional medium or compound. After that, cells were incubated with CCK‐8 for 1 h at 37 °C and the absorbance value was measured at 450 nm. Z‐VAD‐FMK (HY‐16658B), Rapamycin (HY‐10219), 3‐Methyladenin (HY‐19312), and Necrostatin‐1 (HY‐15760) were purchased from MCE. Necroptosis Inducer Kit with TSZ (C1058S) was purchased from Beyotime Biotechnology. The related information of the 10 metabolites used in this study is listed in Table S2 (Supporting Information).

### Cell Death Assay

Propidium iodide (PI) staining, tunnel staining, and flow cytometry were used for the cell death assay. In brief, for in vitro assay, cells were washed and resuspended in PBS containing 10 µg mL^−1^ PI and incubated for 20 min at room temperature under dark conditions. The samples were then analyzed by flow cytometry. In order to detect in vivo cell death, the tunnel assay was conducted on paraffin embedded tissue using the Tunnel assay kit HRP‐DAB (Abcam, ab206386). Briefly, the sections were deparaffinized and rehydrated as previously described. Tumor sections were permeabilized with proteinase K, followed by quenching of endogenous peroxidase activity and equilibration using a Terminal deoxynucleotidyl Transferase (TdT) buffer. Apoptotic cells were labeled using a TdT enzyme and incubated for 2 h at room temperature, followed by a 30‐min incubation with streptavidin‐HRP conjugate. HRP‐positive cells were developed using diaminobenzidine (DAB). All images were captured with a microscope (Leica, DFC420C).

### Animal Models

All animal experimental protocols were approved by the Medical Experimental Animal Care Commission of Ren Ji Hospital (RA‐2021‐192). The investigation was conducted in accordance with the ethical standards and according to the national and international guidelines as well as the Committee for Ethical Review of Research Involving Animal Subjects at Ren Ji Hospital (approval number: RA‐2021‐192). To investigate the effect of TauT on ferroptosis of PCa cells, twenty 8‐week‐old male nude mice were divided into two groups (*n* = 10 per group) to subcutaneously inject with DU145+sgCTRL or DU145+sgTauT cells (1 × 10^7^) respectively. One week after inoculation, for each group, DMSO (*n* = 5) or RSL3 (*n* = 5) were injected intratumorally at a dose of 5 mg k^−1^g every two days for 3 weeks. At the end of the treatment, mice were euthanized and the subcutaneous tumors were excised for histological examination.

To evaluate the effects of M2 polarized macrophages on tumor anti‐ferroptosis activity in vivo, 1 × 10^7^ DU145 cells were mixed in a 4:1 ratio with the M0 macrophages pre‐incubated with either DU145‐con EVs or DU145‐181oe EVs. The relevant cell mixture was named as conEV‐mix or 181oeEV‐mix respectively. Similar to the above assay, 20 mice were divided into two groups (*n* = 10 per group) to subcutaneously inject with conEV‐mix or 181oeEV‐mix respectively. One week later, for each group, DMSO (*n* = 5) or RSL3 (*n* = 5) were injected intratumorally at the same dose to above every two days for 3 weeks. After 3 weeks, the mice were euthanized, and the subcutaneous tumors were removed and fixed in formaldehyde for hematoxylin and eosin (H&E) staining and IHC assay.

### Statistical Analysis

Statistical and image analyses were performed using GraphPad Prism software (version 9.5). Data are expressed as the mean ± SD of at least three independent experiments. Statistical tests are reported in the figure legends, *p* < 0.05 was considered significant. Expression of SLC6A6 in single cell subpopulations was calculated from The Tumor Immune Single Cell Center (TISCH) in prostate cancer dataset (accession number: GSE141445).^[^ ^41^ ^]^ The LXRα ChIP‐seq data used in this study was obtained from the hTFtarget database (http://bioinfo.life.hust.edu.cn/hTFtarget#!/) and corresponds to the GSE77039 dataset. The potential target gene of miR‐181a‐5p was predicted by miRDB (https://mirdb.org).

## Conflict of Interest

The authors declare no conflict of interest.

## Author Contributions

H.X. and X.D. contributed equally to this work. Y.‐X.F., W.‐Q.G., and B.D. conceived the concept; H.X, X.D, Z.T., and N.J. performed all experiments and data analyses; H.X. and Y.‐X.F. contributed to original manuscript drafting; H.X., Y.‐X.F., W.‐Q.G., and B.D. contributed to manuscript editing.

## Supporting information

Supporting Information

## Data Availability

The data that support the findings of this study are available from the corresponding author upon reasonable request.
