# Supplementary material for: Taurine Inhibits Ferroptosis Mediated by the Crosstalk between Tumor Cells and Tumor‐Associated Macrophages in Prostate Cancer
Source: Adv Sci (Weinh). 2023 Nov 29;11(3):2303894. doi: 10.1002/advs.202303894 (PMC10797466; doi:10.1002/advs.202303894)
Supplement: Supplementary file 1 — Supporting Information [file ADVS-11-2303894-s001.pdf]

ADVANCED SCIENCE
Open Access

Supporting Information

for *Adv. Sci.*, DOI 10.1002/advs.202303894

Taurine Inhibits Ferroptosis Mediated by the Crosstalk between Tumor Cells and Tumor-Associated Macrophages in Prostate Cancer

Huixiang Xiao, Xinxing Du, Zhenkeke Tao, Nan Jing, Shijia Bao, Wei-Qiang Gao, Baijun Dong* and Yu-Xiang Fang**

Supporting Information

Taurine inhibits ferroptosis mediated by the crosstalk between tumor cells and tumor-associated macrophages in prostate cancer

Huixiang Xiao, Xinxing Du, Zhenkeke Tao, Nan jing, Shijia Bao, Wei-Qiang Gao, Baijun Dong*, Yu-Xiang Fang**

Xiao et.al Fig S1

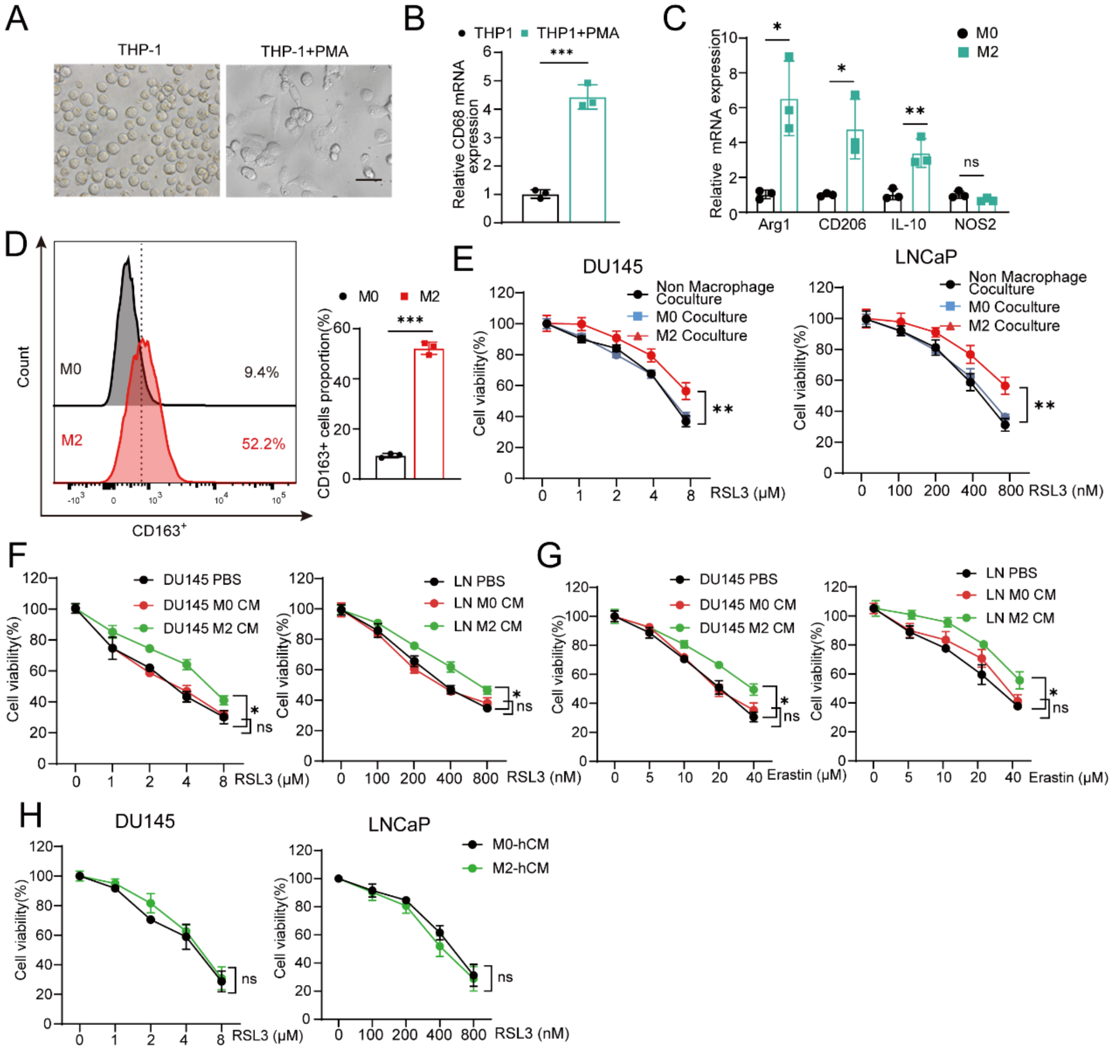

Figure S1. M2 macrophage supernatant possesses an inhibiting effect on ferroptosis in

PCa cells. (A) Representative image of THP-1 cells transformed macrophages after treatment with PMA for 24 h (scale bar= 50 μM). (B) qPCR analysis of the expression of the macrophage marker CD68. (C, D) The expression of ARG1, CD206, IL-10, iNOS (C) and typical M2 marker CD163 (D) in M0/M2 macrophage. (E) Cell viability of PCa cells with or without macrophages co-culture for 24h, followed by treatment with RSL3 for an additional 24h. (F, G) Analysis of cell viability of PCa cells after incubated with M0 CM or M2 CM for 24h following with the RSL3 (F) or Erastin (G) treatment for another 24h. (H) Analysis of cell viability of PCa cells after incubated with M0-hCM or M2-hCM (high molecular weight components conditional medium) for 24h following with the RSL3 treatment for 24h. Each experiment was performed in triplicate and independently repeated three times. (Two-tailed Student's t-test was used for the statistical analysis: ns, not significant; *, $p<0.05$; **, $p<0.01$; ***, $p<0.001$. Data are presented as means ± SD, n=3)

Xiao et.al Fig S2

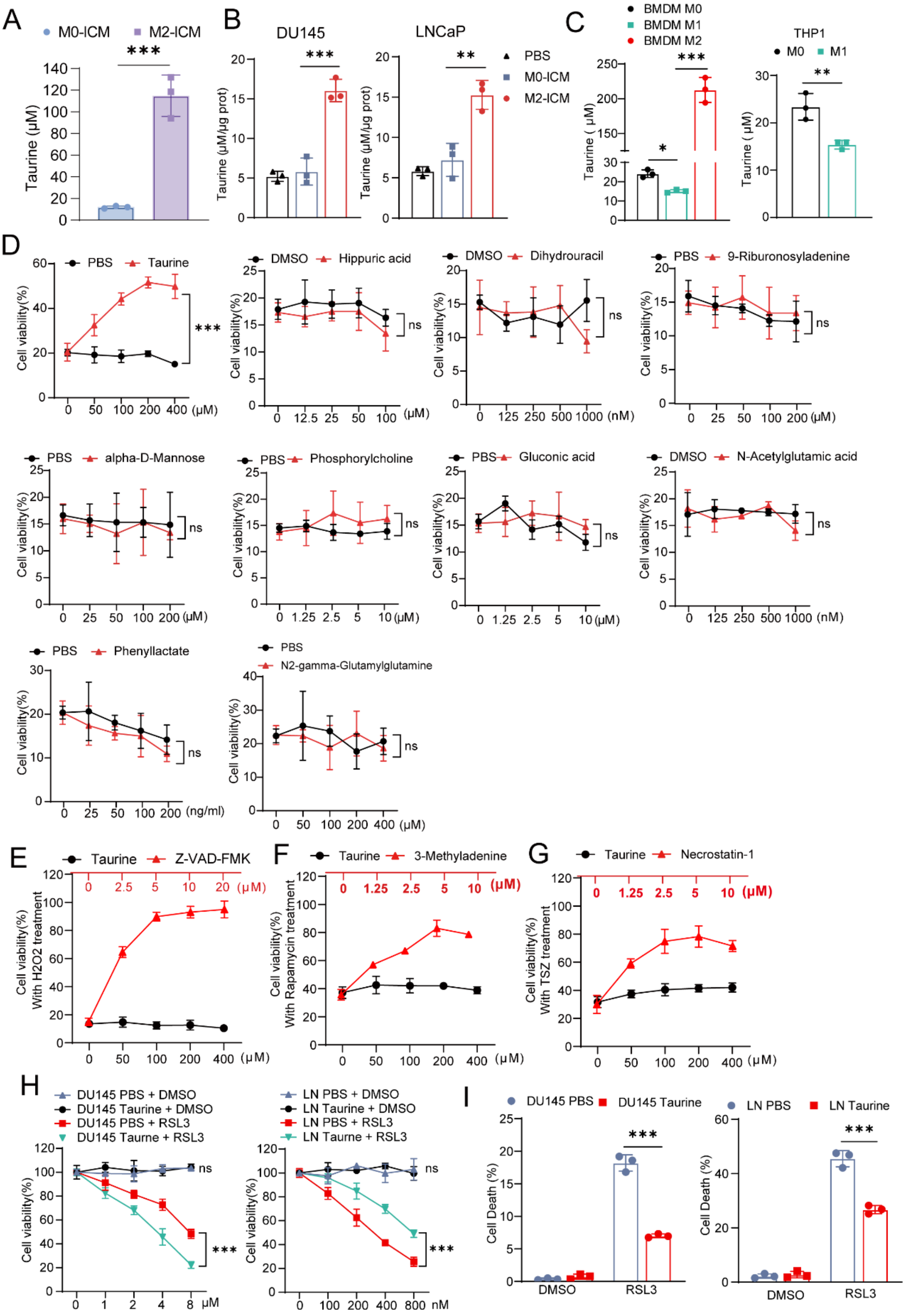

Figure S2. M2 macrophages secreted taurine to inhibit ferroptosis. (A) The taurine level in M0-lCM and M2-lCM. (B) Taurine level in PCa cells after incubated with

M0-lCM or M2-lCM for 24h. (C) The taurine content in BMDM derived M0, M1 or M2 macrophages and in THP1-derived M0 or M1 macrophages. (D) Cell viability of DU145 cells treated with each metabolite in a series of concentration gradients for 24h followed by RSL3 treatment (8 μM) for 24 hours. (E) Cell viability of DU145 cells treated with H2O2 (300 μM) followed with in a series of concentration gradients taurine or Z-VAD-FMK (apoptosis inhibitor, as a positive-control). (F) Cell viability of DU145 cells treated with rapamycin (500 nM) followed with in a series of concentration gradients taurine or 3-methyladenine (autophagy inhibitor, as a positive-control). (G) Cell viability of DU145 cells treated with TSZ (20 ng/ml TNF + 0.2 μM SM-164 + 20 μM Z-VAD-FMK, necroptosis inducer) followed with in a series of concentration gradients taurine or necrostatin-1 (necroptosis inhibitor, as a positive-control). (H) Cell viability of PCa cells treated with or without taurine, followed by the treatment with DMSO or with RSL3 for 24h. (I) The cell death of PCa cells treated with PBS or taurine, followed with DMSO or with RSL3 (4 μM for DU145 cells and 400 nM for LNCaP cells) treatment for 24h. Each experiment was repeated independently three times, and each treatment was replicated three times. (Two-tailed Student's t-test was used for the statistical analysis: ns, not significant; *, $p<0.05$; **, $p<0.01$; ***, $p<0.001$. Data are presented as means ± SD, n=3)

Xiao et.al Fig S3

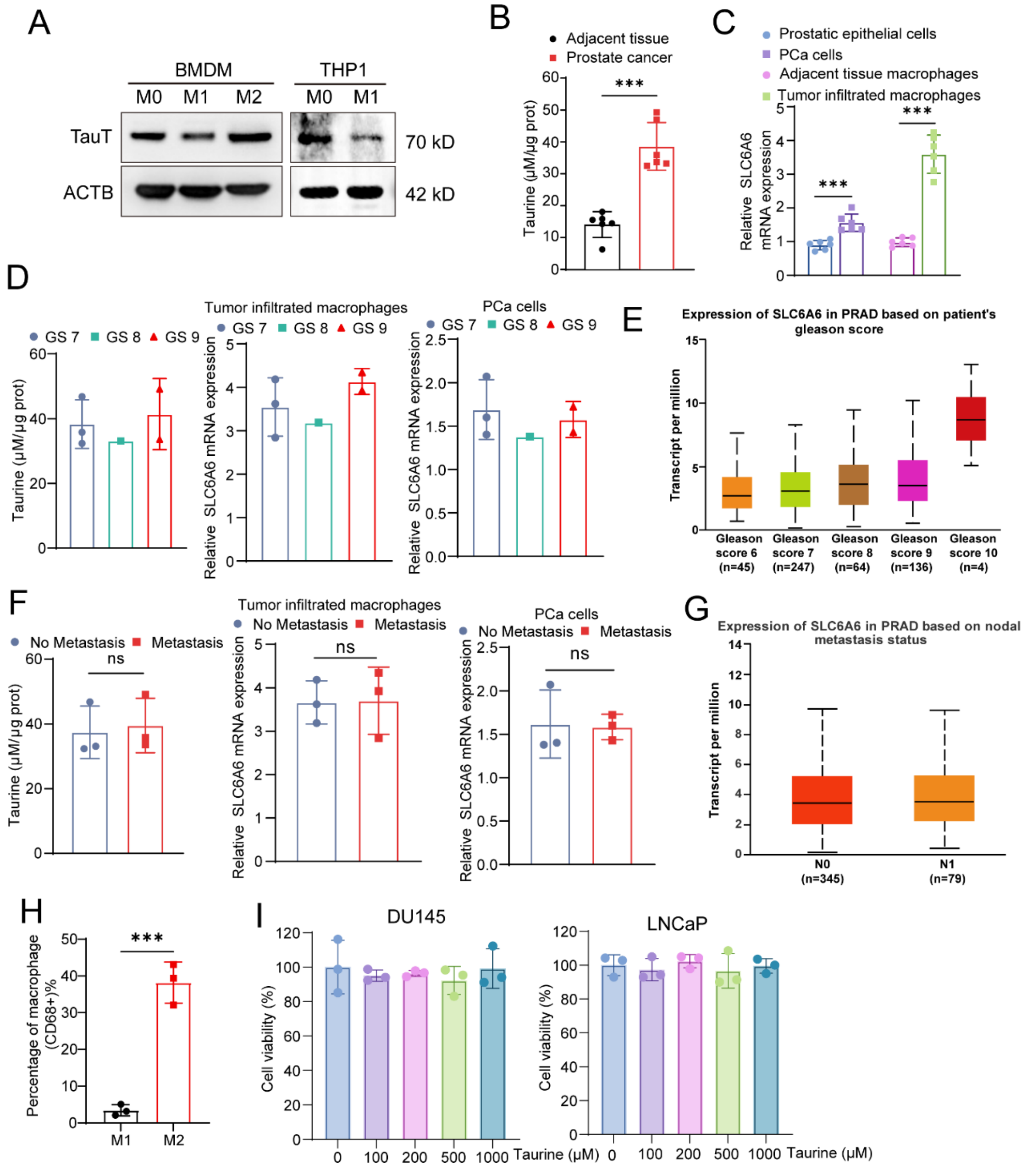

Figure S3. Elevated expression of TauT in M2 macrophages increases export of taurine. (A) The protein expression of TauT in BMDM derived M0, M1 or M2 macrophages and in THP1 derived M0 or M1 macrophages. (B) Taurine level in PCa patient derived cancer tissue and adjacent normal tissue (n=6). (C) The SLC6A6 expression was significantly upregulated in both tumor-infiltrated macrophages and tumor cells in PCa tumor tissues (n=6). (D) Taurine content and TauT expression in

macrophages or tumor cells have no significant difference among tumor tissues with variant Gleason Scores. (E) Analysis of the TauT expression in tumor samples with variant Gleason Scores in the TCGA PRAD database. (F) Taurine content and TauT expression in macrophages or tumor cells have no significant difference among tumor tissues with or without metastasis. (G) Analysis of the TauT expression in tumor samples with or without metastasis in the TCGA PRAD database. (H) The percentage of M1 and M2 macrophages in PCa tissue samples. (I) Cell viability of PCa cells after treated with taurine for 72h in variant concentration. Each experiment was performed in triplicate and independently repeated three times. (Two-tailed Student's t-test was used for the statistical analysis: **, $p<0.01$; ***, $p<0.001$. Data are presented as means ± SD, n=3)

Xiao et.al Fig S4

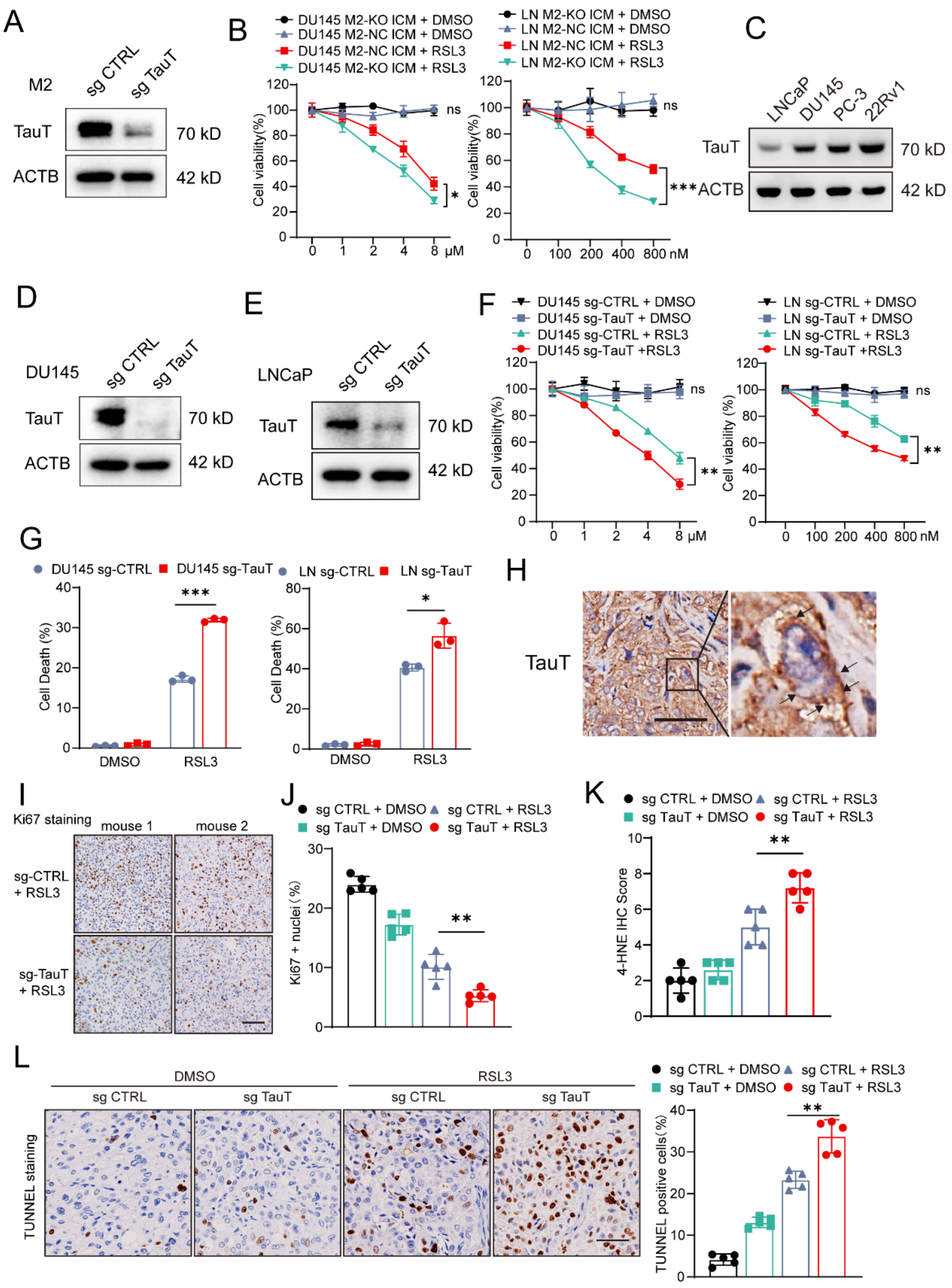

Figure S4. Knock out TauT promotes ferroptosis in PCa cells. (A) Confirmation of the TauT expression in M2 macrophages after TauT knockout. (B) Cell viability of PCa cells treated with M2-KO lCM or M2-NC lCM, followed by the treatment with DMSO or with RSL3 for 24h. (C) The endogenous TauT expression in LNCaP,

DU145, PC3 and 22Rv1 cells. (D, E) Confirmation of the TauT expression DU145 cells, and LNCaP cells after TauT knockout. (F) Cell viability of Taut KO or CTRL PCa cells treated with taurine (100 μM), followed by the treatment with DMSO or with RSL3 for 24h. (G) The cell death of Taut KO or CTRL PCa cells treated with taurine (100 μM), followed with DMSO or with RSL3 (4 μM for DU145 cells and 400 nM for LNCaP cells). (H) Representative IHC images of the subcellular location of the TauT. (Scale bar = 50 μM). (I) Representative IHC images of Ki67 staining of sg-CTRL + RSL3 group and sg-TauT + RSL3 group described in Figure 3I. (Scale bar = 100 μM) (J) The calculation of Ki67 + nuclei in relevant groups described in Figure 3I. (K) The 4-HNE score in relevant groups described in Figure 3I. (L) Representative IHC images of Tunnel staining and the calculation of Tunnel positive cells in relevant groups described in Figure 3I. (Scale bar = 50 μM). Each experiment was performed in triplicate and independently repeated three times. (Two-tailed Student's t-test was used for the statistical analysis: **, $p<0.01$; ***, $p<0.001$. Data are presented as means ± SD, n=3)

Xiao et.al Fig S5

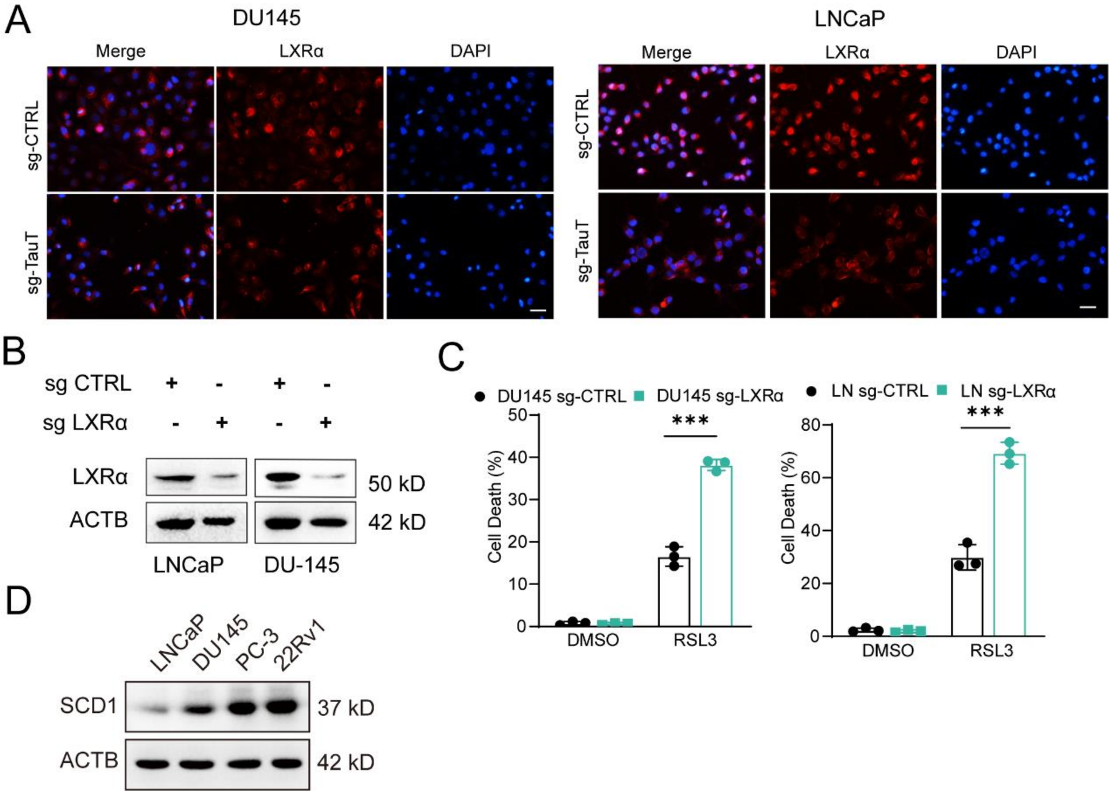

Figure S5. Taurine promotes LXRα nuclear translocation for anti-ferroptosis. (A) IF staining of LXRα in PCa cells with or without TauT knockout. (Scale bar = 50 Mm). (B) The protein expression of LXRα in LNCaP and DU145 cells after LXRα knockout. (C) The cell death of LXRα KO or CTRL PCa cells treated with taurine (100 μM), followed with DMSO or with RSL3 (4 μM for DU145 cells and 400 nM for LNCaP cells). (D) The endogenous SCD1 expression in LNCaP, DU145, PC3 and 22Rv1 cells. Each experiment was performed in triplicate and independently repeated three times. (Two-tailed Student's t-test was used for the statistical analysis: **, $p<0.01$; ***, $p<0.001$. Data are presented as means ± SD, n=3)

Xiao et.al Fig S6

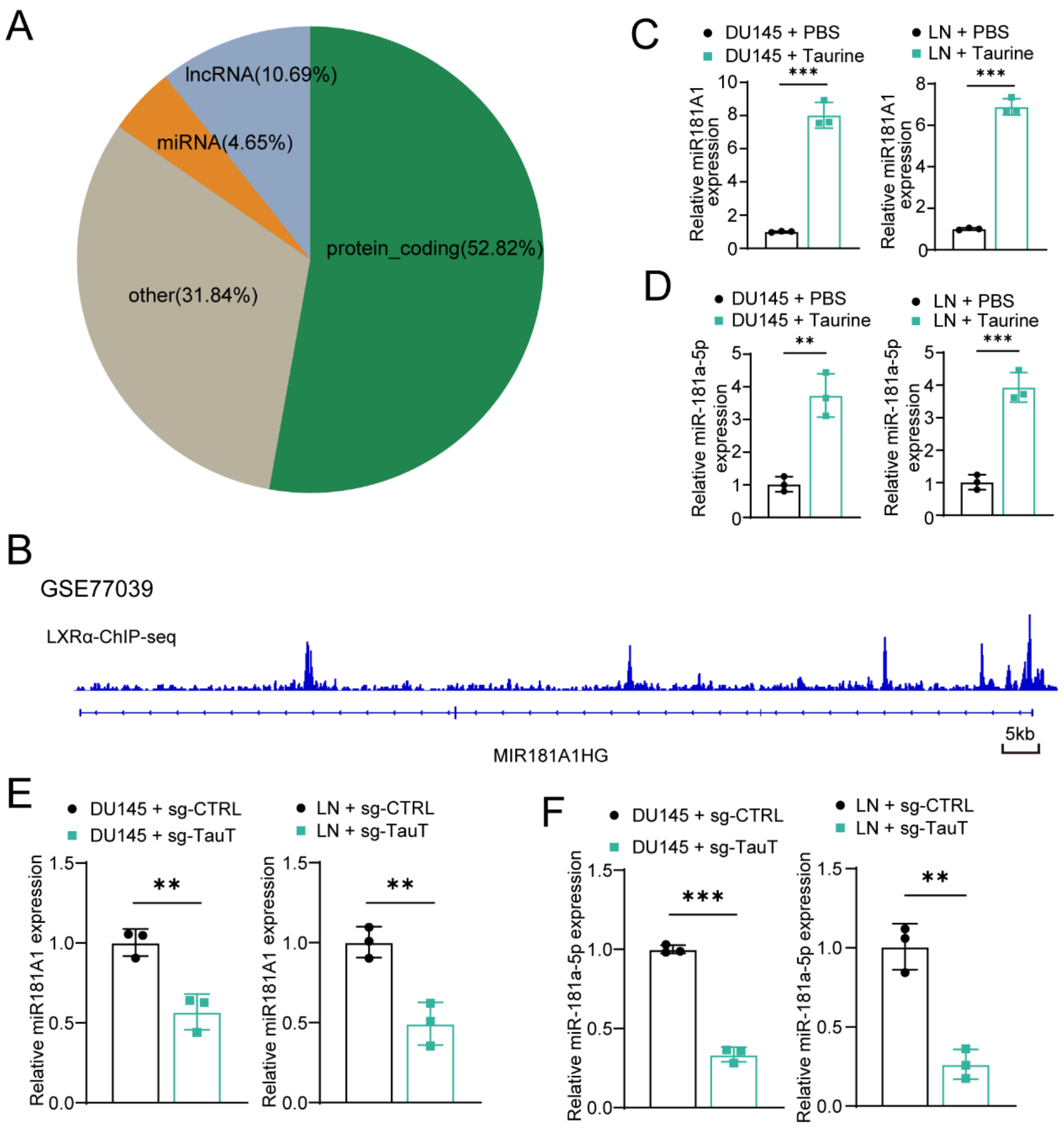

Figure S6. miR-181a-5p was a potential target of LXRα. (A) Pie chart showing the proportion of gene types potentially regulated by LXRα, based on the analysis of GSE77039 dataset. (B) Prediction of miR-181a-5p as a potential target of LXRα by ChIP-seq data analysis using dataset from GSE77039. (Scale bar = 5 kb). (C, D) The expression of miR-181A1 (C) and miR-181a-5p (D) in PCa cells treated with or with taurine. (E, F) The expression of miR-181A1 (E) and miR-181a-5p (F) in TauT KO or CTRL PCa cells with treatment of taurine (100 μM). Each experiment was performed in triplicate and independently repeated three times. (Two-tailed Student's t-test was

used for the statistical analysis: **, p<0.01; ***, p<0.001. Data are presented as means ± SD, n=3)

Xiao et.al Fig S7

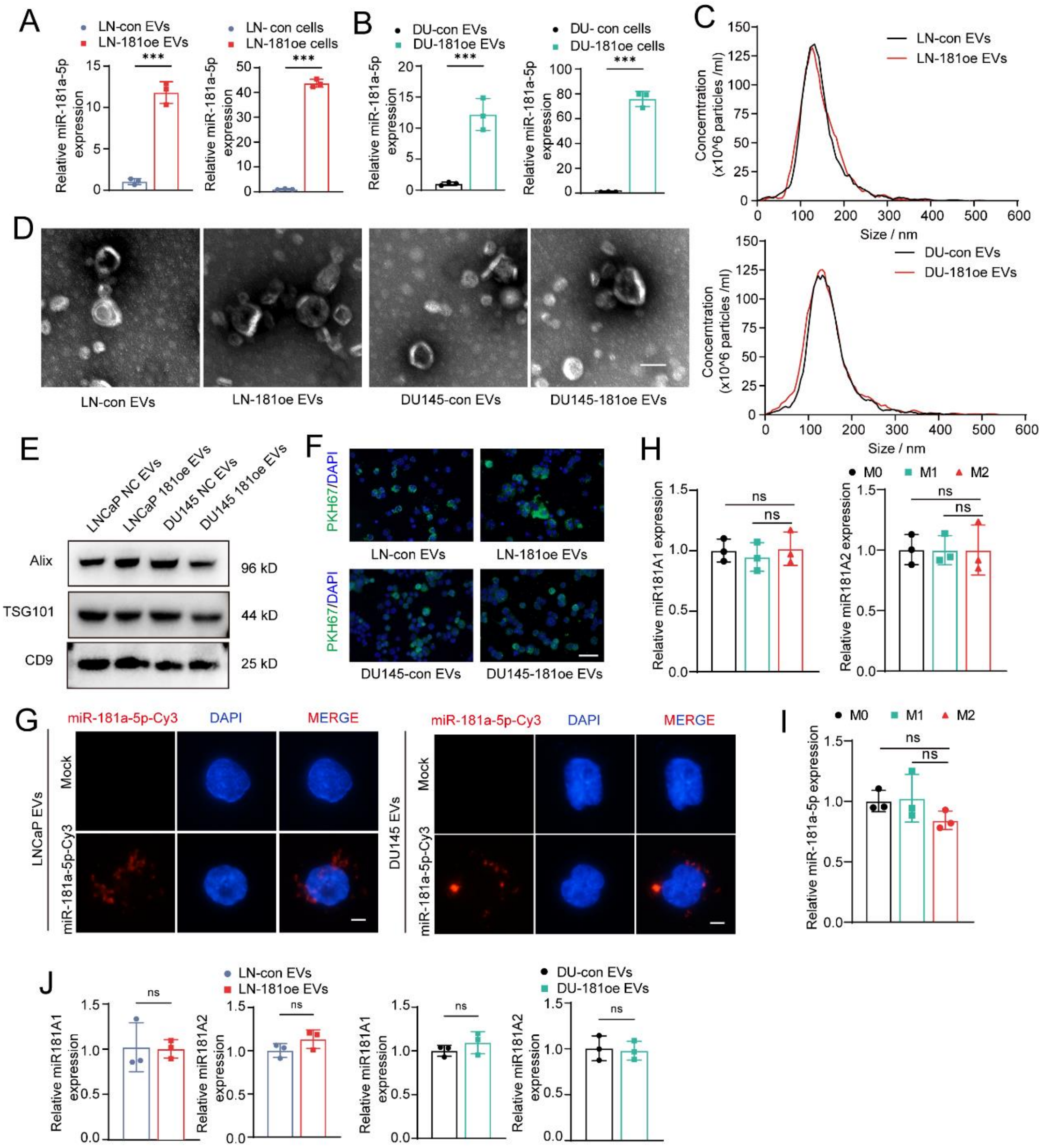

Figure S7. The characteristics of EVs are similar between con-EVs and 181-oe EVs. (A, B) The EV and intracellular expression of mature miR-181a-5p in LNCaP (A) and DU145 (B) cells after stable overexpression of miR-181a-5p. (C, D) Character analysis of relevant EVs by using Nano Sight nanoparticle tracking analysis (C) and electron microscopy (D) (Scale bar = 100 nM). (E) The expression of EV markers

CD9, Alix, and Tsg101 in relevant EVs. (F) PKH67-staining (green) tracing assay for detection of the intake of relevant EVs in macrophages (Scale bar = 20 μM). (G) Representative image of miR-181a-5p tracing in macrophages. (scale bar = 5 μM). (H, I) The expression of both miR-181A1 and miR-181A2 (H), and miR-181a-5p (I) in THP1 derived M0, M1 or M2 macrophages. (J) The expression of both miR181A1 and miR181A2 in macrophages after intake of DU-181oe EVs vs. DU-con EVs or LN-181oe EVs vs. LN-con EVs. Each experiment was performed in triplicate and independently repeated three times. (Two-tailed Student's t-test was used for the statistical analysis: ns, not significant; ***, $p<0.001$. Data are presented as means ± SD, n=3)

Xiao et.al Fig S8

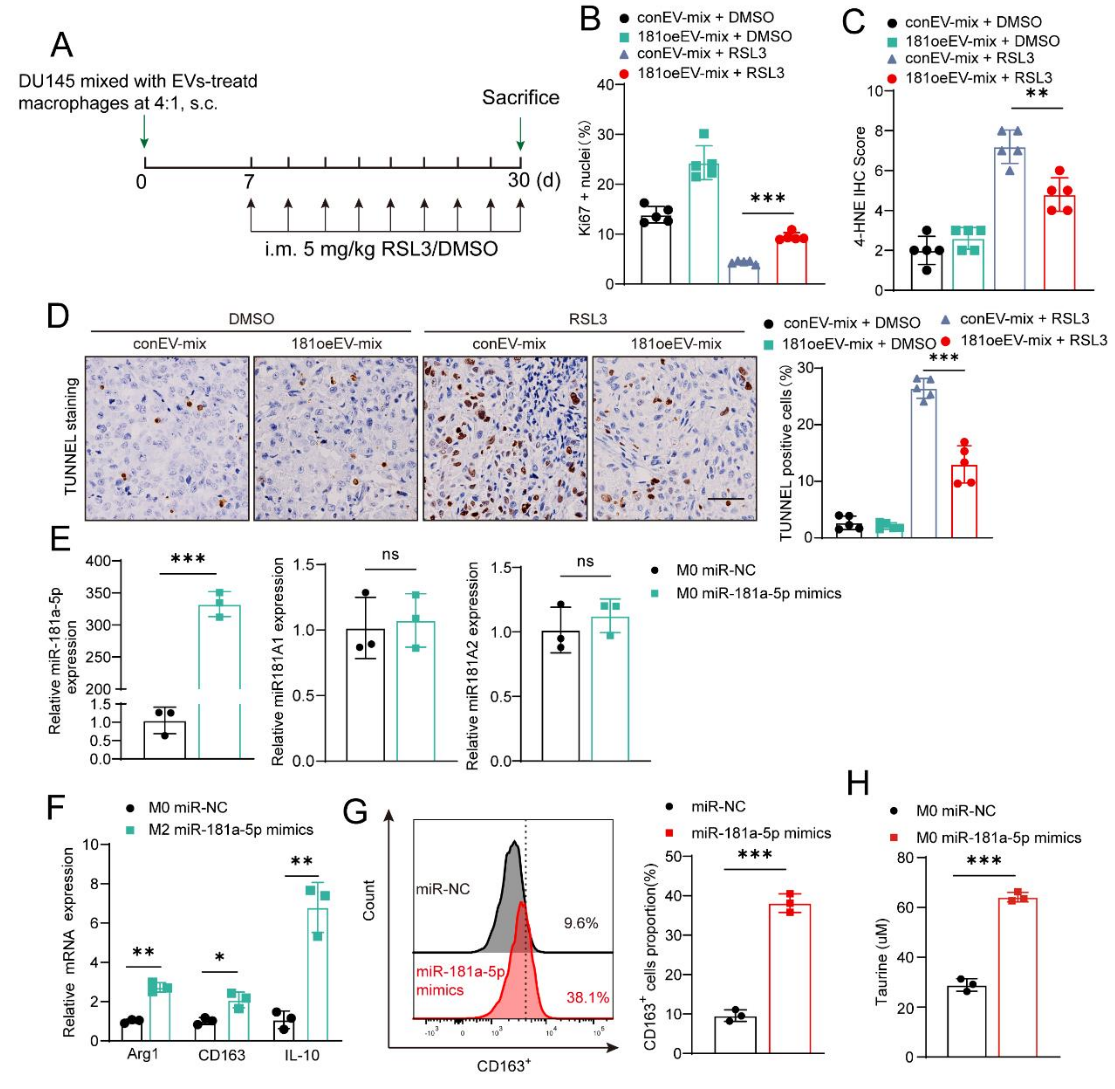

Figure S8. Direct overexpression of mir-181-5p in macrophages promotes M2 polarization. (A) Schematic illustration of experimental design about anti-ferroptosis evaluation in vivo. DU145 cells were mixed with macrophages (tumor cells : macrophages = 4:1) which were pre-treated with DU145-con EVs or DU145-181oe EVs to inject subcutaneously into nude mice. (B) The calculation of Ki67 + nuclei in relevant groups described in Figure 6G. (C) The 4-HNE score in relevant groups described in Figure 6G. (D) Representative IHC images of Tunnel staining and the calculation of Tunnel positive cells in relevant groups described in Figure 6G. (Scale bar = 50 μM). (E) The expression of miR-181a-5p, miR181A1 and miR181A2 in M0 macrophage after overexpression of miR-181a-5p mimics. (F, G) qRT-PCR assay (F) and flow cytometry analysis (G) for expression of typical M2 markers in macrophages after overexpression of miR-181a-5p mimics. (H) taurine level of M0 macrophages after overexpression of miR-181a-5p mimics. Each experiment was performed in triplicate and independently repeated three times. (Two-tailed Student’s t-test was used for the statistical analysis: ns, not significant; *, $p<0.05$; **, $p<0.01$; ***, $p<0.001$. Data are presented as means ± SD, n=3)

Xiao et.al Fig S9

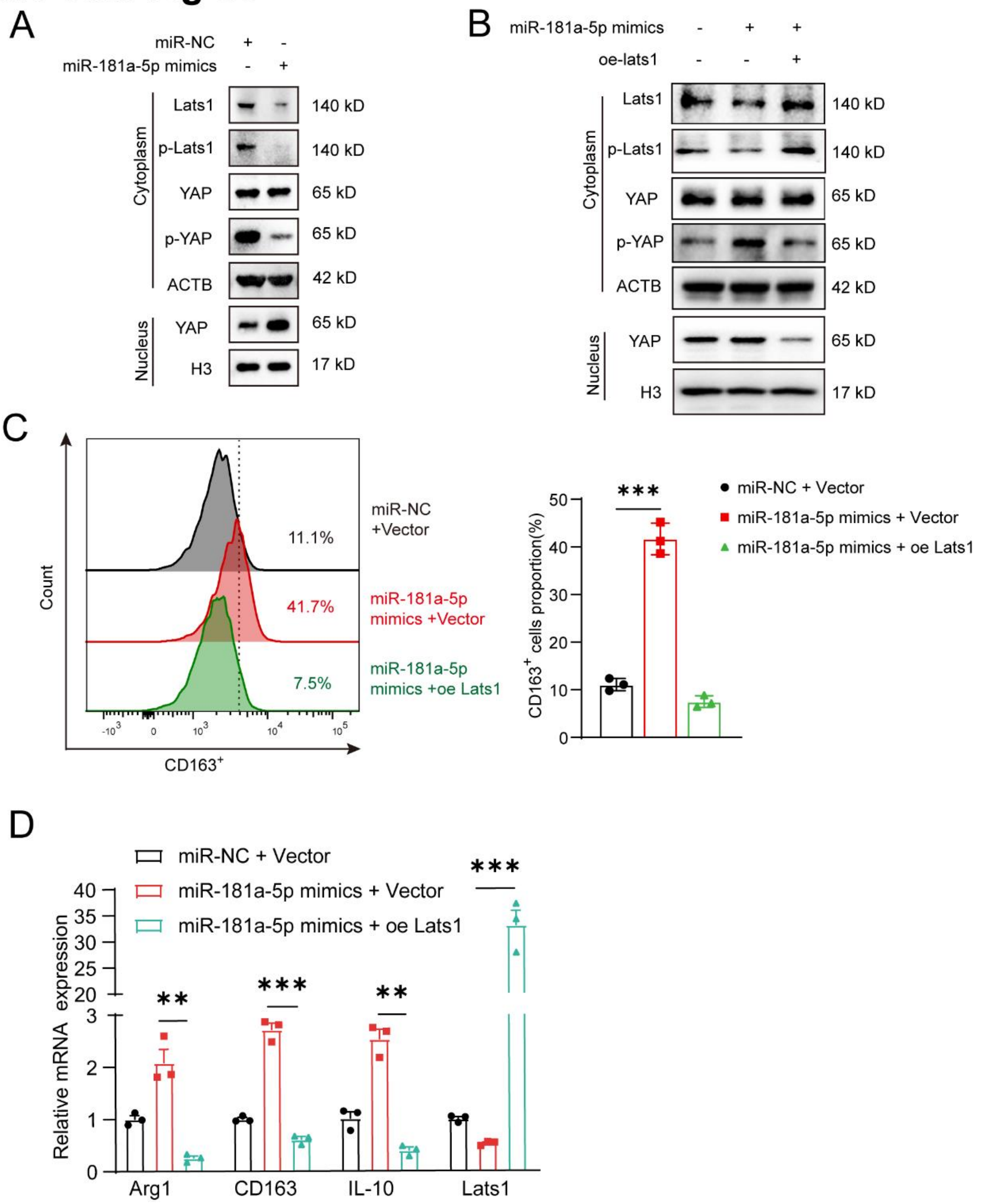

Figure S9. MiR-181a-5p inhibits the Hippo pathway via targeting Lats1. (A) The expression of Hippo pathway and its downstream YAP in M0 macrophages after overexpression of miR-181a-5p mimic vs. miR-NC mimic. (B) The expression of Hippo pathway and its downstream YAP in miR-181a-5p mimic overexpressed M0 macrophages after further overexpression of lats1 CDS as a rescue assay. (C) Typical image by flow cytometry analysis and check of CD163+ cells proportion in miR-181a-5p mimic overexpressed M0 macrophages after further overexpression of

lats1 CDS as a rescue assay. (D) The expression of M2 macrophage markers in miR-181a-5p mimic overexpressed M0 macrophages after further overexpression of lats1 CDS as a rescue assay. Each experiment was performed in triplicate and independently repeated three times. (Two-tailed Student's t-test was used for the statistical analysis: **, $p<0.01$; ***, $p<0.001$. Data are presented as means ± SD, n=3)

Table S1 Top 10 differential metabolite identified by non-target metabolomics

Metabolites	log2(FC_M2/M0)	p-value
Hippuric acid	6.912	0.005
N2-gamma-Glutamylglutamine	4.709	0.001
9-Riburonosyladenine	3.704	0.001
Taurine	2.448	0.001
N-Acetylglutamic acid	1.993	0.006
alpha-D-Mannose	1.847	0.009
Gluconic acid	1.816	0.007
Phenyllactate	1.794	0.017
Dihydrouracil	1.636	0.015
Phosphorylcholine	1.046	0.023

Table S2 10 metabolites physiological concentration in human and product information

Metabolites	Dose concentration[1]	Identifier	Source	Ref.	Concentration used in Fig.2B
Hippuric acid	16.74 ± 11.16 μM	S5618	Selleck	[1]	25 μM
N2-gamma-Glutamylglutamine	117.00 μM	L302213	Aladdin	[2]	100 μM
9-Riburonosyladenine	73 μM	BCP20755	Biochem partner	[3]	75 μM
Taurine	93.0 ± 35.7 μM	T8691	Sigma	[4]	100 μM
N-Acetylglutamic acid	200 ± 30 nM	S6245	Selleck	[5]	250 nM
alpha-D-Mannose	64.0 ± 12.0 μM	HY-N7389B	MCE	[6]	50 μM
Gluconic acid	3.295 ± 0.534 μM	S3595	Selleck	[7]	2.5 μM
Phenyllactate	43.2 ± 14.3 ng/mL	P113801	Aladdin	[8]	50 ng/ml
Dihydrouracil	310 nM	E0750	Selleck	[9]	300 nM
Phosphorylcholine	2.2 ± 1.0 μM	HY-B2233B	MCE	[10]	2.5 μM

1. Data was obtained from The Human Metabolome Database.

Table S3. Antibodies information

Antibodies	Brand	Catalog No.	Dilution and dosage
FITC anti-human CD45	Biolegend	982316	1:100 for Flow
APC anti-human EpCAM	Biolegend	324207	1:100 for Flow
PE/Cyanine7 anti-human CD11b	Biolegend	982608	1:100 for Flow
PerCP/Cyanine5.5 anti-mouse CD68 Antibody	Biolegend	137009	1:100 for Flow
PE anti-human CD163	Biolegend	333605	1:100 for Flow
Brilliant Violet 605 anti-human CD86 Antibody	Biolegend	374213	1:100 for Flow
Lats1	CST	3477S	1:1000 for WB
p-Lats1	CST	9157	1:1000 for WB
YAP1	CST	14074S	1:1000 for WB
p-YAP1	CST	13008	1:1000 for WB
TauT	Abclonal	A14783	1:1000 for WB<br>1:200 for IHC
SCD1	Abclonal	A16429	1:1000 for WB<br>1:200 for IHC
CD163	abcam	ab182422	1:1000 for WB<br>1:200 for IHC
LXRα	ProteinTech	14351-1-AP	1:1000 for WB<br>1:100 for IF
FUS	ProteinTech	11570-1-AP	1:1000 for WB
β-actin	ProteinTech	66009-1-Ig	1:5000 for WB
Histone-H3	ProteinTech	17168-1-AP	1:5000 for WB
Non-phospho (Active) YAP (Ser127) (E6U8Z) Rabbit mAb	CST	29495	1:1000 for WB
Tead4	Abcam	ab58310	1:100 for ChIP
Flag	Sigma	F1804	1:100 for ChIP
Normal rabbit IgG	CST	2729S	1:200 for ChIP
4-HNE	abcam	48506	1:200 for IHC
Ki67	abcam	ab15580	1:200 for IHC
Donkey α -rabbit Alexa Flour 594	Invitrogen	A21207	1:400 for IF
HRP-conjugated Affinipure Goat Anti-Mouse IgG(H+L)	ProteinTech	SA00001-1	1:5000 for WB

HRP-conjugated Affinipure Goat Anti-Rabbit IgG(H+L)	ProteinTech	SA00001-2	1:5000 for WB

Table S4 Clinical information of prostate cancer patient

	case #	Age	Gleason Score	TNM
Used for tested taurine content and TauT expression	1	63	4+3=7	T2cN1M0
	2	63	4+5=9	T2cN0M1
	3	72	4+3=7	T2cN0M0
	4	76	3+4=7	T2cN0M1
	5	71	4+5=9	T3bN0M1
	6	70	4+4=8	T2cN0M0
Used for checked polarization state of macrophages	7	74	3+4=7	T3aN1M1
	8	74	3+4=7	T2bN0M1
	9	74	5+4=9	T2cN0M0

Table S5 Primers used in RT-PCR

Gene	Primer-F	Primer-R
CD68	GGAAATGCCACGGTTCATCCA	TGGGGTTCAGTACAGAGATGC
CD163	CCAGAAGGAACTTGTAGCCACAG	CAGGCACCAAGCGTTTTGAGCT
Arg1	GTGGAAACTTGCATGGACAAC	AATCCTGGCACATCGGGAATC
IL10	TCAAGGCGCATGTGAACTCC	GATGTCAAACTCACTCATGGCT
Nos2	TCTATGTTTGCGGGGATGTG	GTCCTTCTTCGCCTCGTAAG
Lats1	CACTGGCTTCAGATGGACACAC	GGCTTCAGTCTGTCTCCACATC
Actb	CACCATTGGCAATGAGCGGTTC	AGGTCTTTGCGGATGTCCACGT
Scd	CCTGGTTTCACTTGGAGCTGTG	TGTGGTGAAGTTGATGTGCCAGC
Fus	CAGACAGGGAAACTGGCAAGCT	GGCGAGTAGCAAATGAGACCTTG
miR-181A1	TTCAACGCTGTCGGTGA	GAACATGTCTGCGTATCTC
miR-181A2	AACATTCAACGCTGTCGGTG	GAACATGTCTGCGTATCTC
Slc6a6	TGATGTGGCTGAGTCAGGTCCT	CTGGCTATCCAGTCCAAGCAAG

Table S6 Primers used for ChIP-qPCR

Primer ID	Primer-F	Primer-R
LXRα-SCD BS1	ATGTGGATCACCTGAGGTCAG	TCAAGAAATTCTCCTGCCTCA
LXRα-SCD BS2	CAGGGACAGATCAGTAGGGTC	AATAACTTGCCCATGGTCACA

LXRα-SCD BS3	CCAAGCCCTTTCGCCTGCTGC	GATGTTTTGGAGATTCTTCAGA
LXRα-181A1 BS1	ATGCCTTTTAATTATTTGTAA	GTATAATAGGGATGTTGTGAT
LXRα-181A1 BS2	CATAAAAATGCATAAAATATA	TCCAAACTCACCGACAGCGTT
LXRα-FUS BS1	GTTTCACCATGTTGGCCAG	ATAAAGCTGACCGGGTGCGGT
LXRα-FUS BS2	CGAAATCCCTGCTGTCTTTCA	CTGCGCCCTGAGGTTGACTTCG
Neg-CTRL	GGCCGGAGCTTCTCGAACTA	CTTGCAGCCTCAGCCCTTC
TEAD4-ARG1 BS1	GCATACAAAGAACTTTCAGGATGTG	ATGGAGATTTGCTAACTTTC
TEAD4-ARG1 BS2	AGCCTATGTTGGCAACGGGT	TACCATGTGTCCGATGCAGTTC
TEAD4-CD163 BS1	GTACGGGTAGGGAGGCAGTA	TCAACAAAGGCACCCCAGTT
TEAD4-CD163 BS2	CTTTTTGAGTTGACTCCGCCT	TAAATCTTCTTGTATTATTCCCTAGAAATG
TEAD4-SLC6A6 BS1	GGCGTCTGAGGAGTTTTTGC	AGAGGAGTGACTGGGTCTCC

Supplementary Reference cited in Table S2:

1. Duranton, F., et al., *Normal and pathologic concentrations of uremic toxins.* J Am Soc Nephrol, 2012. **23**(7): p. 1258-70.
2. Hammond, J.W., et al., *gamma-Glutamylglutamine identified in plasma and cerebrospinal fluid from hyperammonaemic patients.* Clin Chim Acta, 1990. **194**(2-3): p. 173-83.
3. Uri, A., et al., *A new class of compounds, peptide derivatives of adenosine 5'-carboxylic acid, includes inhibitors of ATP receptor-mediated responses.* Bioorg Med Chem, 1994. **2**(10): p. 1099-105.
4. Trabado, S., et al., *The human plasma-metabolome: Reference values in 800 French healthy volunteers; impact of cholesterol, gender and age.* PLoS One, 2017. **12**(3): p. e0173615.
5. Yoshimi, N., et al., *Blood metabolomics analysis identifies abnormalities in the citric acid cycle, urea cycle, and amino acid metabolism in bipolar disorder.* BBA Clin, 2016. **5**: p. 151-8.
6. C. Lentner ; West Cadwell, N.J., *Geigy scientific tables*. 8th Rev edition ed. Medical education Div. 1981–1992, Basel, Switzerland: Ciba-Geigy Corp.
7. Koike, S., et al., *A snapshot of plasma metabolites in first-episode schizophrenia: a capillary electrophoresis time-of-flight mass spectrometry study.* Transl Psychiatry, 2014. **4**(4): p. e379.
8. Oezguen, N., et al., *Serum 3-phenyllactic acid level is reduced in benign multiple*

sclerosis and is associated with effector B cell ratios. Mult Scler Relat Disord, 2022. **68**: p. 104239.

9. Jiang, H., et al., *Measurement of endogenous uracil and dihydrouracil in plasma and urine of normal subjects by liquid chromatography-tandem mass spectrometry.* J Chromatogr B Analyt Technol Biomed Life Sci, 2002. **769**(1): p. 169-76.

10. Ilcol, Y.O., et al., *Choline status in newborns, infants, children, breast-feeding women, breast-fed infants and human breast milk.* J Nutr Biochem, 2005. **16**(8): p. 489-99.
